# SARS-CoV-2-encoded small RNAs are able to repress the host expression of SERINC5 to facilitate viral replication

**DOI:** 10.3389/fmicb.2023.1066493

**Published:** 2023-02-16

**Authors:** Salvador Meseguer, Mari-Paz Rubio, Begoña Lainez, Beatriz Pérez-Benavente, Raúl Pérez-Moraga, Sergio Romera-Giner, Francisco García-García, Olalla Martinez-Macias, Antonio Cremades, Francisco J. Iborra, Oscar Candelas-Rivera, Fernando Almazan, Enric Esplugues

**Affiliations:** ^1^Molecular and Cellular Immunology Laboratory, Centro de Investigación Príncipe Felipe (CIPF), Valencia, Spain; ^2^Bioinformatics and Biostatistics Unit, Centro de Investigación Príncipe Felipe (CIPF), Valencia, Spain; ^3^Hospital Universitario de la Ribera, Valencia, Spain; ^4^Biological Noise and Cell Plasticity Laboratory, Centro de Investigación Príncipe Felipe (CIPF), Associated Unit to Instituto de Biomedicina de Valencia-CSIC, Valencia, Spain; ^5^Molecular and Cellular Biology Department, Centro Nacional de Biotecnología (CNB), CSIC, Madrid, Spain; ^6^Department of Comparative Medicine, Yale School of Medicine, New Haven, CT, United States

**Keywords:** SARS-CoV-2, viral miRNAs, SERINC5, MAVS, innate immune response

## Abstract

Serine incorporator protein 5 (SERINC5) is a key innate immunity factor that operates in the cell to restrict the infectivity of certain viruses. Different viruses have developed strategies to antagonize SERINC5 function but, how SERINC5 is controlled during viral infection is poorly understood. Here, we report that SERINC5 levels are reduced in COVID-19 patients during the infection by SARS-CoV-2 and, since no viral protein capable of repressing the expression of SERINC5 has been identified, we hypothesized that SARS-CoV-2 non-coding small viral RNAs (svRNAs) could be responsible for this repression. Two newly identified svRNAs with predicted binding sites in the 3′-untranslated region (3’-UTR) of the SERINC5 gene were characterized and we found that the expression of both svRNAs during the infection was not dependent on the miRNA pathway proteins Dicer and Argonaute-2. By using svRNAs mimic oligonucleotides, we demonstrated that both viral svRNAs can bind the 3’UTR of SERINC5 mRNA, reducing SERINC5 expression *in vitro*. Moreover, we found that an anti-svRNA treatment to Vero E6 cells before SARS-CoV-2 infection recovered the levels of SERINC5 and reduced the levels of N and S viral proteins. Finally, we showed that SERINC5 positively controls the levels of Mitochondrial Antiviral Signalling (MAVS) protein in Vero E6. These results highlight the therapeutic potential of targeting svRNAs based on their action on key proteins of the innate immune response during SARS-CoV-2 viral infection.

## 1. Introduction

Host restriction factors are a set of cell proteins that limit the replication of viruses at various stages through different mechanisms (Colomer-Lluch et al., 2018). Many host restriction factors are induced in response to type I interferon (IFN-I), whose expression is stimulated in the detection of viral pathogens by the activation of pattern recognition receptors (PRRs), such as retinoic acid-induced gene I (RIG-I) and melanoma differentiation-associated protein 5 (MDA5). Serine incorporator protein 5 (SERINC5) is a member of a protein family that participates in lipid biosynthesis and/or transport in mammalian cells (Inuzuka et al., 2005). Although it is not induced by IFN-I, SERINC5 has also been considered a restriction factor since it impairs the infectivity of several retroviruses, such as murine leukemia virus (MLV), human immune deficiency virus (HIV), equine infectious anemia virus (EIAV) (Ahi et al., 2016; Chande et al., 2016; Trautz et al., 2017), and other viruses (Ahi et al., 2016; Firrito et al., 2018). To date, there is limited knowledge of the mechanism of action of the SERINC5 protein (Matheson et al., 2015). Incorporation of this protein into HIV-1 virions has been shown to block the formation of the virus-cell fusion pore, preventing virus entry into new target cells (Matheson et al., 2015; Rosa et al., 2015; Usami et al., 2015). On the other hand, two recent studies have shown two additional antiviral activities for SERINC5. One describes that SERINC5 inhibits Hepatitis B virion (HBV) secretion by interfering with the glycosylation of HBV envelope proteins (Liu et al., 2020), and the other demonstrates that SERINC5 can interact with the outer mitochondrial antiviral signaling protein (MAVS) and the E3 ubiquitin ligase/adaptor protein TRAF6, resulting in MAVS aggregation and polyubiquitination of TRAF6. These events are critical for IFN-I signaling and nuclear factor kappa B (NFkB) activation (Zeng et al., 2021).

Conversely, viruses have developed different strategies to antagonize most of the host restriction factors (Goujon et al., 2013; Kane et al., 2013; Simon et al., 2015; Colomer-Lluch et al., 2018; Ghimire et al., 2018). In the case of SERINC5, its antiviral functions are counteracted by several virus-encoded proteins including, HIV-1 Nef, the glycogag protein of MLV, and the EIAV S2 protein. These viral proteins alter the subcellular localization of SERINC5 and prevent its insertion into viral particles (Chande et al., 2016). For instance, HIV-1 Nef decreases levels of SERINC5 at the plasma membrane and relocates it into the lysosomal compartments to avoid its incorporation in the HIV-1 virions, thus, facilitating the HIV-1 infectivity (Chande et al., 2016). Moreover, mutations in the HIV-1 envelope glycoprotein present in some strains were shown to alter the sensitivity of the virus to SERINC5 (Usami et al., 2015; Ahi et al., 2016; Chande et al., 2016; Beitari et al., 2017). Although the main reported viral mechanism to antagonize the antiviral activity of SERINC5 is to relocate SERINC5 within the cell, it has been shown that expression of SERINC5 can also be down-regulated upon the infection both *in vitro* and *in vivo* (Li et al., 2020). For instance, the infection by the classical swine fever virus (CSFV), which causes a highly contagious viral disease in pigs (Becher et al., 2003), reduces SERINC5 expression by an unknown mechanism (Li et al., 2020). This fact again supports the key role of SERINC5 in the host defense against viral infection.

It has been proved that both DNA and RNA viral genomes can encode non-coding small viral RNAs (svRNAs) like miRNAs (Skalsky and Cullen, 2010; Nanbo et al., 2021). miRNAs are small (19–28 nucleotides) non-coding single-stranded RNAs that bind to the 3′ untranslated regions (3′ UTR) of target mRNA/s, regulating their stability and translation. These elements can be considered more strategic than viral proteins in terms of regulation of gene expression due to their small size, their absence of immunogenicity and their multi-target hit with rapid evolution capacity. When viral miRNAs are expressed in host cells, they can optimize the cellular environment and promote viral replication and survival by targeting host genes involved in proliferation, apoptosis and immune defense (Skalsky and Cullen, 2010; Kincaid and Sullivan, 2012). It was generally believed that RNA viruses would not encode miRNAs to avoid excision of their genomes or transcriptome by the miRNA processing machinery. However, there are RNA viruses that express small regulatory RNAs such as miRNAs. For example hav-miR-1-5p and hav-miR-2-5p are expressed during Hepatitis A virus (HAV) infection (Shi et al., 2014). Moreover, the deep sequencing analysis of small RNAs from lungs of mice infected with severe acute respiratory syndrome coronavirus (SARS-CoV) revealed three 18-22 nt svRNAs originated from the nsp3 and N genomic regions of SARS-CoV. Authors found that one of them, svRNA-N, contributes to SARS-CoV pathogenesis by regulating the production of proinflammatory cytokines (Morales et al., 2017). Despite these examples in RNA viruses, these small RNAs do not seem to possess the canonical stem-loop structure of miRNAs, and their biogenesis and mechanism of action are not completely clear (Varble and ten Oever, 2011; Mishra et al., 2019).

SARS-CoV-2 is an enveloped positive-sense, single-stranded RNA virus, that belongs to the *Sabecovirus* subgenus (Coronaviridae Study Group of the International Committee on Taxonomy of Viruses, 2020) and is behind the current pandemic of Coronavirus disease 2019 (COVID-19). This virus causes symptoms of the common cold (fever, coughing, etc.), unusual symptoms (loss of smell or taste), breathing problems, and gastrointestinal symptoms (nausea, vomiting, etc.). The condition can change into a serious respiratory illness such as severe pneumonia and acute respiratory distress syndrome (ARDS), finally causing death (Redd et al., 2020; Yang et al., 2020). The RNA viral genome (about 30,000 nt) carries two overlapping open reading frames (ORF 1a and 1b) that encode for the main components of the transcription-replication complex and genes encoding for the structural and genus-specific proteins (S, 3a, 3b, E, M, 6, 7a, 7b, 8, N, and 10) (Hartenian et al., 2020; Mittal et al., 2020; Wu et al., 2020; Zhu et al., 2020). Recently, several studies also demonstrated the existence of miRNAs encoded in the SARS-CoV-2 genome and their biological relevance (Grehl et al., 2021; Pawlica et al., 2021; Singh et al., 2022). Also, by using computational approaches, several studies have predicted the possible existence of viral miRNAs with diverse roles in the pathogenicity of this virus (Abedi et al., 2021).

Based on the key role of SERINC5 in virus infection, we have analyzed the expression of SERINC5 during SARS-CoV-2 infection in two distinct cell lines from a GEO dataset. The *in silico* study revealed a decrease of SERINC5 mRNA during the infection course, suggesting that SARS-CoV-2 antagonizes SERINC5 activity by downregulating its expression. Given that no SARS-CoV-2 protein capable of controlling the expression of SERINC5 has been described so far and considering the emerging evidence pointing to the existence of miRNAs encoded in the genome of the SARS-CoV-2, as occurs in other viruses, we wanted to address the hypothesis that SERINC5 expression can be regulated by svRNAs. Using two different *in silico* approaches, we identified two svRNAs, as putative miRNA-like regulators of SERINC5. We found an anti-correlative expression between these two svRNAs and SERINC5 in different biological samples, including samples from COVID-19 patients. Furthermore, we proved that silencing of both svRNAs during the course of infection of Vero E6 cells restores SERINC5 expression and enhances the levels of its direct interacting partner, MAVS, a master protein involved in antiviral response. We also showed that these molecular changes were accompanied by a reduction in the expression of the viral proteins N and S.

## 2. Materials and methods

### 2.1. Human samples and ethics statement

Swabs and saliva samples were provided by Hospital Universitario de la Ribera (Valencia, Spain). All samples were collected from COVID-19 patients or healthy subjects and written informed consent was obtained from the participants. All procedures were approved by the Ethics Committee of Hospital Universitario de la Ribera (Valencia, Spain) and performed under the guidelines set forth by the Declaration of Helsinki.

### 2.2. Biosafety

All the experiments with SARS-CoV-2 were approved by the National Centre for Biotechnology (CNB-CSIC) Institutional Biosafety Committee (IBC) and were carried out in an appropriate biosafety level 3 (BSL3) laboratory at CNB following the safety guidelines and procedures approved for this kind of laboratory.

### 2.3. Cells and viruses

Vero E6 cells (African green monkey kidney epithelial cells) were obtained from the American Type Culture Collection (ATCC; CRL-1586). HEK293T-hACE2 cells, expressing the human angiotensin I converting enzyme 2 (ACE2), were kindly provided by Dr. Martinez-Sobrido (Texas Biomedical Research Institute, San Antonio, United States).

Vero E6 negative control cells and Vero E6 cells overexpressing SERINC5 were obtained by transfection of Vero E6 cells with 1 μg/mL of the empty plasmid pIRES2 ZsGreen1 or the plasmid pIRES2 ZsGreen1 containing the *Chlorocebus* SERINC5 cDNA, using Lipofectamine 2000 reagent (Invitrogen) and Opti-MEM medium according to manufacturer’s instructions. Forty-eight hours after transfection, cells were sorted by ZsGreen signal using SONY sorter and selected with 1 mg/mL G-418 for 48 h and then grown with 0.5 mg/mL G-418.

In all cases, cells were cultured in high glucose Dulbecco’s modified Eagle medium (DMEM, Gibco) supplemented with 25 mM HEPES, 10% Fetal Bovine Serum (FBS), 1 mM sodium pyruvate, 100 U/mL penicillin, 100 μg/mL streptomycin, 2 mM glutamine and 1 mM non-essential amino acids (growth medium). They were kept at 37°C in a humidified atmosphere with 5% CO_2_.

SARS-CoV-2 MAD6 isolate was kindly provided by Dr. Luis Enjuanes (CNB-CSIC, Madrid, Spain). This virus was obtained in March 2020 from the nasal sample of a COVID-19 patient hospitalized in Hospital 12 de Octubre (Madrid, Spain), after obtaining the patient’s informed consent and Regional Government permits. The genome sequence is identical to that of Wuhan-Hu-1 (GenBank MN908947) except for three mutations: C3037T (silent), C14408T (P214L in Nsp12), and A234303G (D614G in S). From the nasal sample, the virus was cloned by plaque assay and amplified in confluent Vero E6 cells to generate a working virus stock. This virus stock was used to infect Vero E6 and HEK293T-hACE2 cells using virus growth medium (growth medium containing 2% FBS). A multiplicity of infection (MOI) of 1 plaque forming unit (PFU) per cell was used in most of the experiments.

### 2.4. Virus titration

Confluent monolayers of Vero E6 cells seeded in 12-well plates were infected with 300 μL of serial 10-fold dilutions of the virus in virus growth medium for 1 h at 37°C. After viral adsorption, the viral inoculum was removed and the cells overlaid with 2 mL virus growth medium containing 1% DEAE-Dextran (Sigma-Aldrich) and 0.6% low-melting-point agarose. After 3 days of incubation at 37°C, the cells were fixed with 10% formaldehyde for 1 h at room temperature, the overlaid removed, and the viral plaques visualized by staining with 0.1% crystal violet in 20% methanol. Visible plaques were counted and virus titer was calculated as PFU/mL.

### 2.5. Plasmids construction

The plasmid pIRES2-ZsGreen 1 (PT3824–5, Clontech) was used to insert the *Chlorocebus* SERINC5 cDNA into its NheI and EcoRI sites. On the other hand, to clone the 3′ end of the 3′UTR of SERINC5 gene, a 1Kb PCR product insert was purified using the PCR Clean-Up kit. Linearization of pMir was performed with MluI according to the manufacturer’s instructions. The purified PCR insert was cloned into linearized pMir with the In-Fusion HD Cloning Plus enzyme mix and then transformed into the provided Stellar Competent Cells. Both plasmid constructs were verified by DNA sequencing. The oligonucleotides used to amplify the full cDNA and the 3’UTR of SERINC5 are indicated in Table 1.

### 2.6. Cell transfections

We used two classes of oligonucleotides in transfection experiments. mirVana miRNA mimics are oligonucleotides designed for their use in *in vitro* and *in vivo* gain-of-function experiments. mirVana miRNA mimics are small, double-stranded RNAs that mimic endogenous precursor miRNAs (pre-miRNAs). One strand is identical to and effectively mimics a known mature miRNA. The manufacturer’s design of these oligonucleotides and their chemical modifications optimize selection of the active strand for uptake and activation by the RNA-induced silencing complex (RISC). On the other hand, mirVana miRNA inhibitors are designed for their use in *in vitro* and *in vivo* loss-of-function experiments. mirVana miRNA inhibitors are single-stranded RNA-based oligonucleotides that are designed to bind to, and inhibit the activity of endogenous miRNAs when introduced into cells. The design coupled to chemical modifications improves potency and specificity for miRNA inhibition.

In particular, we used the custom version of mirVana miRNA mimics and inhibitors since they are synthesized by the manufacturer basing on the unpublished mature miRNA sequences provided by the customer (svRNA 1: ACTCATGCAGACCACACAAGGCAG; svRNA2: CAAAACATTCCCACCAACAGAGCC) (Table 1). Previously, the sequence input passes the design requirements established by manufacturer’s design tool (GeneAssist™ miRNA Workflow Builder). Both, custom mimics and inhibitors, incorporates the same chemical modifications as the manufacturer’s predesigned mirVana mimics and inhibitors. The complete sequences from custom mirVana miRNA mimics, inhibitors or their respective controls require a confidential disclosure agreement. 1–100 nM is the concentration range recommended by the manufacturer for optimization experiments. We observed maximum effects at 60 nM.

For the transfections with the above oligonucleotides, Vero E6 or HEK293T-hACE2 cells were seeded at 500,000 cells/well in 6 well plates. After 24 h, transfection mix for each well was prepared by adding drop by drop with a 100 μL-pipette a mix containing 2.4 μL of 50 μM of one of the above oligonucleotides [mimic molecules (custom mirVana miRNA mimic; Thermofisher) of svRNA 1 (pre-svRNA 1), svRNA 2 (pre-svRNA 2), or antisense oligonucleotides (custom mirVana miRNA inhibitor; Thermofisher) targeting svRNA 1 (anti-svRNA 1), svRNA 2 (anti-svRNA 2) or their respective negative controls (NC) [mirVana miRNA Mimic Negative Control #1 (NC-pre-svRNA; 4,464,058, Thermofisher) and mirVana™ miRNA Inhibitor Negative Control #1 (NC-anti-svRNA; 4,464,076, Thermofisher)]] and 250 μL of Opti-MEM medium to a mix containing 4 μL of Lipofectamine 2000 reagent (Invitrogen) and 250 μL of Opti-MEM medium. After 30 min of incubation, the 500 μL-transfection mix was added to the cells from the well in which the growth medium was previously replaced by 1.5 mL of Opti-MEM medium. The medium was replaced by fresh growth medium 6 h after transfection and cells were infected 24 h after transfection with SARS-CoV-2 at a MOI of 1 UFP/cell.

The same conditions were used for the transfection of HEK293T-hACE2 cells with Sigma siRNAs targeting Dicer (SASI-Hs01-00160748, SASI_Hs01_00130221) or Ago2 (SASI-Hs01-00343736, SASI_Hs01_00161740) or with negative control (NC) siRNA (SIC001).

### 2.7. RNA isolation and RT-qPCR

Total RNA from saliva, from preservation solution in contact with the swab or from cell pellet was isolated using TRI reagent (Sigma) following the manufacturer’s protocol.

To quantify mRNA levels, one-step RT-qPCRs were performed in an Applied Biosystems Step-One Real-Time PCR System. To that end, 25 ng of total RNA were reverse-transcribed and amplified by qPCR in a 12 μL total volume reaction containing specific primers (Table 1), Power SYBR Green PCR Master Mix, MultiScribe Reverse Transcriptase, and RNase Inhibitor (all from Applied Biosystems), according to the manufacturer’s instructions. The amplification efficiency values were very close to 100%. Relative quantitation of mRNA levels was calculated using the ΔΔCt method and ribonuclease P/MRP subunit p30 (RPP30) mRNAs as endogenous control. The viral titer of each sample was estimated by determining the viral E mRNA copies/mL. They were calculated by interpolation in a standard curve (Ct vs. amount) prepared from a serial dilution of a SARS-CoV-2 genome standard [1.05 × 10^8^ genome equivalents/mL (NR-52285, bei Resources)].

**Table 1:** Oligonucleotides used in the study.

Oligonucleotides used for mRNA level quantitation (RT-qPCR)				
Name	Sequence	Gene	Provider	
IFNβ Fwd	CATGAGCTACAACTTGCTTGG	IFNβ	IDT	
IFNβ Rev	TCCTCCTTCTGGAACTGCTG			
ISG20 Fwd	TGACAAGTTTGCCCTGAGTG	ISG20	IDT	
ISG20 Rev	ATGCTTTAACTGGCGTCACC			
CCL20 Fwd	GCTTTGATGTCAGTGCTGCTAC	CCL20	IDT	
CCL20 Rev	TTGGATTTGCGCACACAG			
SERINC5 Fwd	ATCGAGTTCTGACGCTCTGC	SERINC5	IDT	
SERINC5 Rev	GCTCTTCAGTGTCCTCTCCAC			
RPP30 Fwd	CTATTAATGTGGCGATTGACCGA	RPP30	IDT	
RPP30 Rev	TGAGGGCACTGGAAATTGTAT			
Sequences used to order the custom TaqMan miRNA assays				
Name	Target sequence	Gene	Provider	
svRNA 1	ACTCATGCAGACCACACAAGGCAG	svRNA 1	Thermo Fisher Scientific	
svRNA 2	CAAAACATTCCCACCAACAGAGCC	svRNA 2	Thermo Fisher Scientific	
TaqMan miRNA control assay				
Name	Assay ID	Gene	Provider	
U6 snRNA	001973	U6 snRNA	Thermo Fisher Scientific	
Oligonucleotides used for the cloning of the 3’ UTR of SERINC5 into pMIR				
Name	Sequence	Restriction enzyme	Gene	Provider
SERINC5_3UTR_F	GAAacgcgtTGATATCGGCGGTCCCCT	*MluI*	SERINC5	IDT
SERINC5_3UTR_R	GAAacgcgtTTGCACACCACAGATATATATCT	*MluI*		
Oligonucleotides used for the cloning of SERINC5 cDNA into pIRES2 ZsGreen1 plasmid				
Name	Sequence	Restriction enzyme	Gene	Provider
mSERINC5 Fwd	GGACGAgctagcATGTCAGCTCAGTGCTGTGCAGGCCAGCT	*NheI*	SERINC5	IDT
mSERINC5 Rev	GTATTAgaattcTCACACAGAGAACTCCCGGGTGGGGCAGCAGA	*EcoRI*	SERINC5	

The lowercase letters indicate restriction enzyme sites introduced for cloning.

For svRNA quantification, 10 ng of total RNA were reverse-transcribed in 15 μL total reaction volume using the MultiScribe reverse transcriptase and custom miRNA-specific stem-loop RT primers (Table 1). Then, 1.33 μL of the reverse transcription reaction was subjected to a custom TaqMan miRNA assay (Table 1), in a total reaction volume of 12 μL, using specific primers and probes for the svRNAs and U6 snRNA (Table 1), according to the manufacturer’s protocol. Expression values were calculated using the ΔΔCt method and U6 snRNA, the most commonly used endogenous control gene in miRNA RT-qPCR assays. When the expression value was calculated with respect to uninfected samples (reference or control), we used the Ct value of the product detected in the PCR reaction (non-specific product) as the Ct value for the reference sample.

### 2.8. Western blot analysis

Cell extracts were prepared in Laemmli sample buffer (2% SDS, 10% glycerol, 5% 2-mercaptoethanol, 0.004% bromophenol blue and 0.0625 M Tris HCl pH 6.8) containing 0.1 mM leupeptin and 1 mM phenylmethanesulphonyl fluoride, and boiled at 95°C for 10 min. Then, 15 μL of lysates were resolved by SDS/PAGE (12% polyacrylamide) and transferred to PVDF membranes (GE Healthcare, Amersham Biosciences) following the manufacturer’s recommendations. Membranes were blocked for 1 h at room temperature with 5% dried skimmed milk in TBS (20 mM Tris–HCl pH 7.5, 150 mM NaCl) and then probed overnight at 4°C with specific antibodies diluted in TTBS (TBS containing 0.1% Tween 20) containing 3% dried skimmed milk. We used the following primary antibodies: 1:5,000-diluted anti-SARS-CoV-2 N protein (40143-MM05, Sino Biological), 1:1,000-diluted anti-SARS-CoV-2 S protein (GTX632604, GeneTex), 1:500-diluted anti-SERINC5 (ab204400), 1:500-diluted anti-MAVS (24,930, Cell Signaling), and 1:10,000-diluted anti-Tubulin (ab6160). The blots were then incubated with the secondary antibodies anti-rabbit (A0545) or anti-mouse (A9044) IgG-horseradish peroxidase-conjugated (Sigma-Aldrich) diluted in TTBS-3% dried skimmed milk for 1 h at room temperature, and the immune complexes were detected using Lumi-light Western Blotting substrate (Roche) or ECL prime western blotting detection system (Amersham), according to the manufacturer’s instructions. Protein bands were quantified by densitometric analysis with an Image Quant ECL (GE Healthcare).

### 2.9. Fluorescence microscopy

Vero E6 cells were cultured on coverslips in 24-well plates. Twenty-four hours post-seeding, cells were rinsed with PBS, fixed with 4% paraformaldehyde in PBS for 20 min at room temperature, washed with PBS, permeabilized with 0.5% Triton X-100 in PBS for 10 min and washed twice with PBS. Then, they were blocked with a solution containing 4% FBS in PBS for 30 min at room temperature, and incubated with 1:100-diluted anti-SERINC5 (ab204400), rabbit anti-MAVS (24,930, Cell Signaling) or mouse anti-MAVS (sc-166,583) in blocking solution for 1 h at room temperature. After three washes with PBS, bound antibodies were detected by incubation, as appropriate, with AlexaFluor 594-conjugated anti-mouse (A11020, Invitrogen) or AlexaFluor 633-conjugated anti-rabbit (A21072, Invitrogen) secondary antibodies in blocking solution for 1 h at 37°C. Slides were mounted in Prolong Gold antifade reagent with DAPI (Molecular Probes, 936,576) and images were obtained with an Apotome-equipped Axio Observer Z1 microscope (Carl Zeiss AG).

### 2.10. Luciferase reporter assay

Vero E6 cells were seeded in 24-well plates at 50,000 cells/well. After 24 h, transfection mix for each well was prepared by adding, drop by drop with a 100 μL-pipette, a mix containing 500 ng of a Firefly Luciferase reporter plasmid, 25 ng of Renilla Luciferase control vector (Promega; internal control), 0.5 μL of 50 μM of one of the svRNA mimics (pre-svRNA 1, pre-svRNA 2) or NC-pre-miR and 50 μL of Opti-MEM medium to a mix containing 1 μL of Lipofectamine 2000 reagent (Invitrogen) and 50 μL of Opti-MEM medium. After 30 min of incubation, the 100 μL-transfection mix was added to the cells from the well in which the growth medium was previously replaced by 400 μL of Opti-MEM medium. The medium was replaced by fresh growth medium 6 h after transfection. 48 h post-transfection, cells were washed with PBS and lysed by shaking the plate/s during 20 min at room temperature with 100 μL of 1X Passive Lysis buffer per well. 20 μL of each cell lysate were transferred into the luminometer plate. Firefly and Renilla luciferase activities from the cell extracts were respectively measured in the luminometer (settings: a 2-s premeasurement delay, followed by a 10-s measurement period for each reporter assay) after sequential addition of 100 μL of Luciferse Assay reagent II and 100 μL of Stop and Glo reagent from the Dual-luciferase Reporter Assay System (Promega).

### 2.11. Reanalysis of deposited sequencing data and selection of svRNA candidates

To analyze SERINC5 expression in Calu3 and Caco2 cells, SRA files were downloaded from GSE148729. We used for this study SRA files from the sequencing of polyA RNA from Calu3 cells and Caco2 cells, infected or not with SARS-CoV and SARS-CoV-2. Normalized read counts from the SRA files were rescaled to avoid negative values and subsequently logarithmic transformed. Expression levels of SERINC5 at the different time points were plotted for each sample collection.

Data from small RNA were collected from GSE148729 and fastq files were downloaded using SRA-Toolkit. Sequences from these files were trimmed with cutadapt. We removed (i) the Truseq adapter for small RNA (TGGAATTCTCGGGTGCCAAGG), (ii) the first three 5′ nucleotides from the reads, (iii) the reads with a phred below 30, (iv) polyA tails and (v) the very short reads. Then, the DEUS tool for R was used to detect unique reads (human and virus) and to perform differential expression (only reads with zero values in the non-infected condition). Sequence annotation [Homo sapiens (human) GRCh38 (hg38)] and SARS-CoV-2 isolate Wuhan-Hu-1 (NC_045512.2) genomes were used as a reference for the blast and clustering of selected similar sequences. svRNA 2 was one of the most represented in number of counts and also one of the most differentially expressed svRNAs (Supplementary Table S1).

svRNA 1 was selected after exploring the intergenic regions of the SARS-CoV-2 isolate Wuhan-Hu-1 (NC_045512.2) genome with RNA central to find similar non-coding RNA sequences in the human genome. A region from pre-miR-431 was found to be highly similar to the region between N and Orf10 genes from the SARS-CoV-2 genome. This result was also confirmed by searching for similarity with miRBase (search sequences: stem-loop sequences, search method: BLASTN, E-value cutoff: 100, maximum number of hits: 100).

Binding sites for svRNA 1 and 2 were found in the 3’UTR of SERINC5 mRNA using the Diana MR-microT tool.

### 2.12. Statistical analysis

Statistical analysis was performed using Student’s *t*-test and was conducted using GraphPad Prism 8 (GraphPad Software, Inc., San Diego, CA). The statistically significant differences between the means were indicated by asterisks (*p < 0.05, **p < 0.01, or ***p < 0.001), and non-significant differences by ns.

## 3. Results

### 3.1. Levels of SERINC5 mRNA are reduced in COVID-19 patients and this reduction is inversely proportional to the level of two svRNAs predicted to bind SERINC5 mRNA

**Figure 1:**
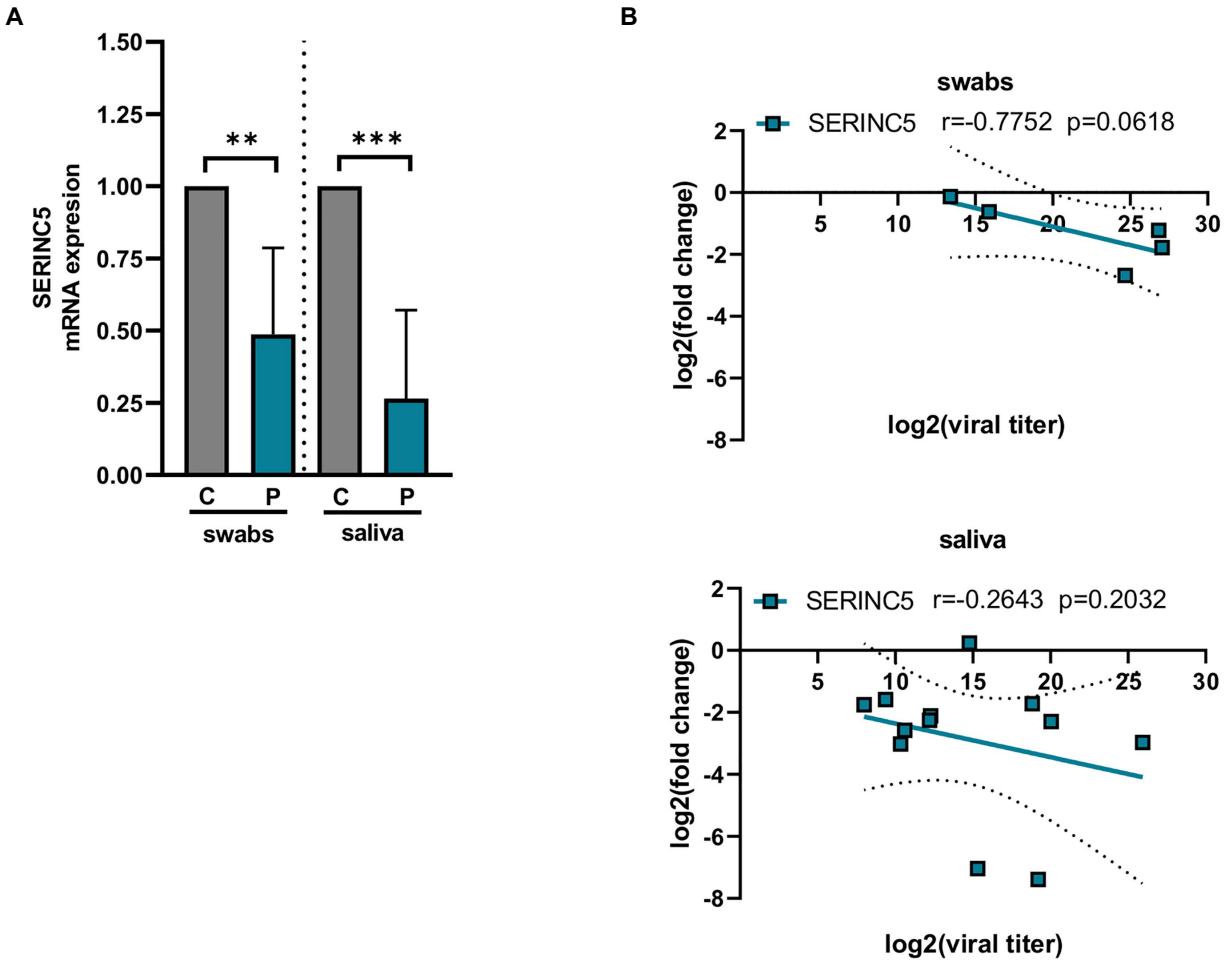
Analysis of the levels of SERINC5 mRNA in nasopharyngeal and saliva samples of COVID-19 patients. **(A)** RT-qPCR analysis of the expression of SERINC5 mRNA in nasopharyngeal (swabs) and saliva samples from COVID-19 patients (P) with respect to healthy patients (C). The control value represents the mean of all control samples. The ΔΔCt method was used for relative quantification using RPP30 mRNA as an endogenous control. Data are represented as log2 fold change with respect to control samples. **(B)** Correlation analysis between log2 fold changes obtained for SERINC5 mRNA and log2 viral titers in swabs (top) and saliva (bottom) samples. Viral titer was expressed as E mRNA copies/mL sample. Differences from control values were found to be statistically significant at *p < 0.05, **p < 0.01, and ***p < 0.001.

SERINC5 was identified as a critical restriction factor for the infectivity of certain viruses such as HIV-1 (Ahi et al., 2016; Chande et al., 2016; Trautz et al., 2017). To explore whether the expression of SERINC5 is affected during SARS-CoV-2 infection, the level of SERINC5 mRNA was analyzed from transcriptomic data of Calu3 cells infected with SARS-CoV-2 deposited in GEO (GSE148729) and from nasopharyngeal and saliva samples from COVID-19 patients. The analysis of GSE148729 data (Supplementary Figure S1) showed that SERINC5 mRNA levels progressively decrease with the time of infection in Calu3 cells. Similar results were obtained with Caco2 cells (data not shown). Then, we evaluated the level of SERINC5 mRNA in nasopharyngeal (swabs) and saliva samples from patients with COVID-19 and controls. RT-qPCR analysis of SERINC5 mRNA levels showed a significant reduction in both types of samples from patients compared to controls (Figure 1A). When the correlation between SERINC5 expression and viral titer (assessed in terms of subgenomic E mRNA expression) was analyzed (Figure 1B), we found a clear inverse correlation in the swabs samples. However, in saliva this correlation was not significant, likely due to the complex nature of this sample type, with many contaminants (proteins, complex organic molecules, and bacteria) and RNAses that may affect the quality of RNA. Altogether, these data indicated that the infection of SARS-CoV-2 induced a reduction in the levels of SERINC5 mRNA.

**Figure 2:**
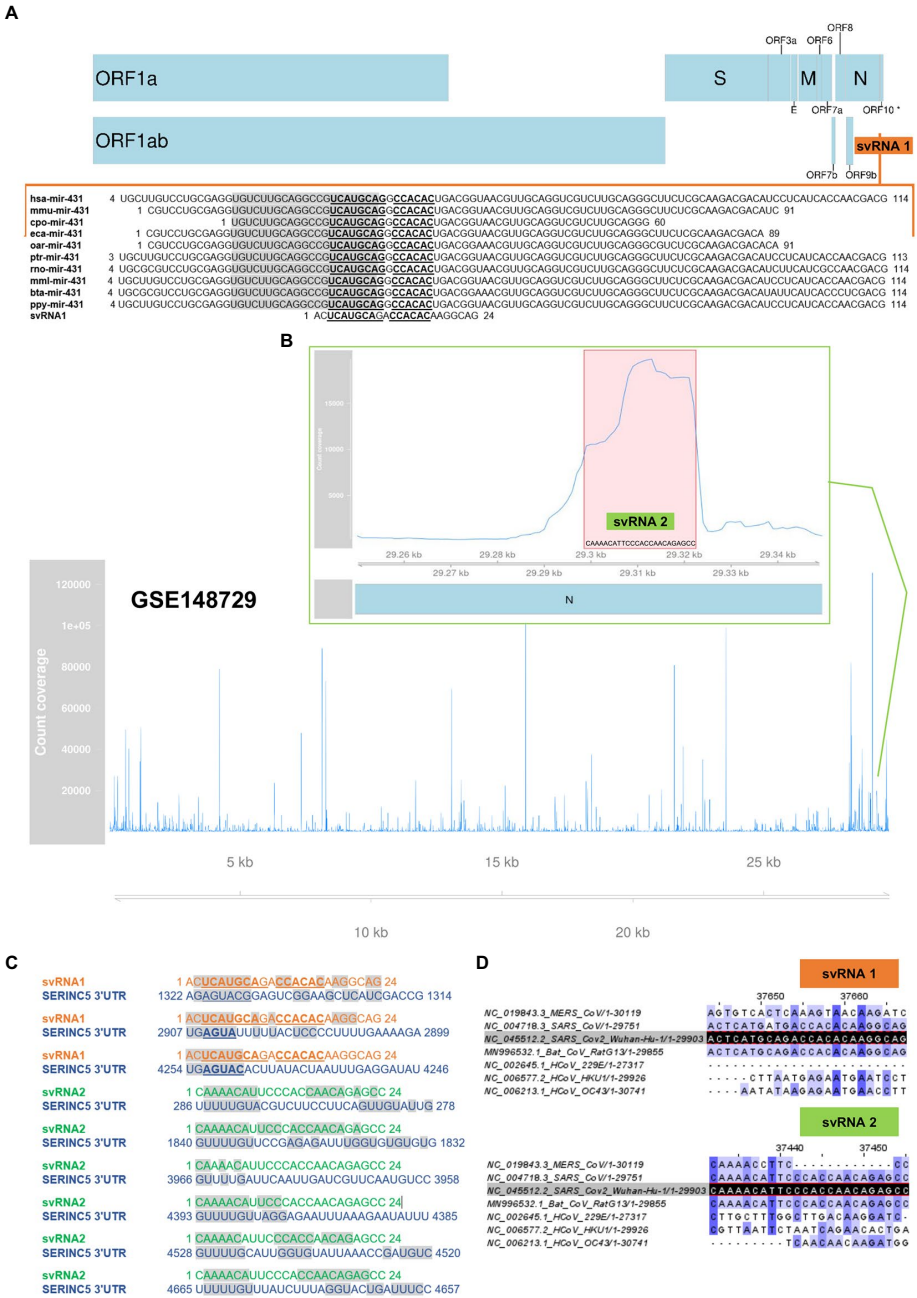
*In silico* prediction of SARS-CoV-2 svRNAs interacting with SERINC5 mRNA. **(A)** Identification of svRNA 1 using RNA Central Resources. Sequence and genomic location of svRNA 1 and alignment of svRNA 1 sequence with those of pre-miRNA-431 from different species are shown. The mature miRNA sequences are in grey shadow and the conserved nucleotides among species are in bold letters. (*) ORF10 has so far little experimental support as a protein-coding gene **(B)** Identification of svRNA 2 by reanalysis of GSE148729 dataset. Count coverage of small RNA sequences from SARS-CoV-2-infected Calu3 cells aligning with the SARS-CoV-2 genome is indicated. The top panel shows the region’s count coverage containing the svRNA 2 at 24 hpi. The red shadow indicates the svRNA 2 sequence. **(C)** Predicted interaction of svRNAs 1 and 2 with the 3’UTR of SERINC5 mRNA according to the Diana MR-microT tool. Grey shadow indicates nucleotides involved in the interaction. **(D)** Alignment of SARS-CoV-2 genome-encoded svRNA 1 and 2 sequences (dark shadow) with the genomes of other coronaviruses: MERS-CoV, SARS-CoV, HCoV-229E, HCoV-HKU1, HCoV-OC43, and the SARS-like betacoronavirus Bat coronavirus RaTG13. A higher intensity of the blue shade denotes higher conservation.

In SARS-CoV-2 no viral protein capable of repressing the expression of SERINC5 in host cells has been identified. Therefore, we investigated the hypothesis that the genome of the virus harbors small RNA regulators of host gene expression, similar to miRNAs, that could be responsible for the reduction of SERINC5 levels during infection. Through an *in silico* study, we identified two svRNAs (svRNAs 1 and 2) with predicted binding sites in the 3’UTR region of the SERINC5 gene (Figure 2). svRNA 1 was identified from the analysis of the intergenic regions of the SARS-CoV-2 genome with the RNA central program. RNA Central Resources can identify, in a sequence query, any small non-coding RNA (sncRNA) sequence similar to those deposited in the database. This database houses all types of ncRNA from a wide range of organisms. RNA central provided a 24 nt sequence, located in the intergenic sequence between N and ORF10 genes at the 3’-end of the SARS-CoV-2 genome (29,534 nt–29,557 nt; Figure 2A), which was similar to the cellular microRNA precursor pre-miRNA-431. This sequence was conserved among several mammalian species, including humans, and showed a binding capacity to the 3’UTR region of SERINC5 mRNA according to the Diana MR-microT tool (Figure 2C). On the other hand, svRNA 2 was selected as one of the most expressed small RNAs from the reanalysis of a small RNA dataset deposited in GEO (GSE148729) that was generated from Calu3 cells infected with SARS-CoV-2 (Figure 2B; Supplementary Table S1). This 24 nt long svRNA mapped in the N gene (29,353 nt–29,376 nt) and its sequence also showed binding sites to the 3’UTR region of SERINC5 mRNA (Figure 2C). Both svRNA candidates were confirmed by the miRNA fold tool, which allows the prediction of microRNA hairpin structures using a genome sequence as Input (Supplementary Table S2). Moreover, the conservation of svRNA sequences among different classes of coronavirus was also analyzed, showing that svRNA 1 and 2 exhibited high conservation grades among coronaviruses SARS-CoV, SARS-CoV-2 and the SARS-like betacoronavirus Bat coronavirus RaTG13, known as the closest relative of SARS-CoV-2 (Figure 2D). Altogether, these *in silico* studies suggested the existence of two SARS-CoV-2 svRNAs predicted to interact with the 3’UTR region of SERINC5 mRNA.

**Figure 3:**
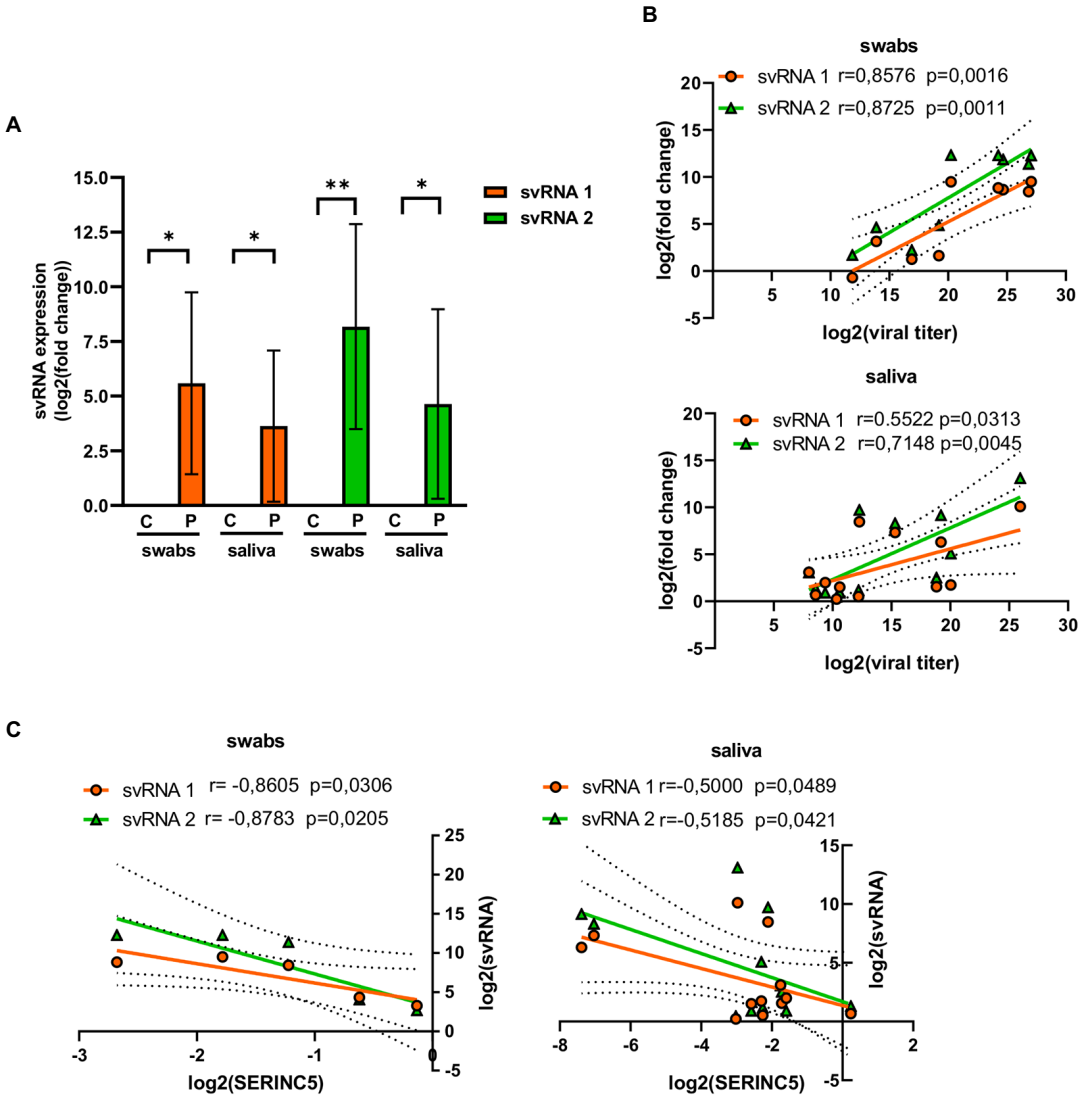
Analysis of the levels of svRNA 1 and 2 in nasopharyngeal and saliva samples of COVID-19 patients. **(A)** RT-qPCR analysis of the expression of svRNA 1 and svRNA 2 in nasopharyngeal (swabs) and saliva samples from COVID-19 patients (P) with respect to control samples (C). The control value represents the mean of all control samples. The ΔΔCt method was used for relative quantification with U6 snRNA as an endogenous control. Data are represented as log2 fold change with respect to control samples. **(B)** Correlation analysis between log2 fold changes obtained for svRNA 1 or svRNA 2 and log 2 viral titers in swabs (top) and saliva (bottom) samples. **(C)** Correlation analysis of log2 fold changes obtained for svRNA 1 or svRNA 2 and SERINC5 mRNA in swabs (left) and saliva (right) samples. Differences from control values were found to be statistically significant at *p < 0.05, **p < 0.01, and ***p < 0.001.

To confirm the existence of svRNA 1 and svRNA 2 in COVID-19 patients, the presence of both svRNAs was analyzed in both nasopharyngeal and saliva samples by RT-qPCR, using specific TaqMan probes (Table 1). Both svRNAs were detected in both types of samples (Figure 3A). Although their levels among the samples were heterogeneous, they showed a significant correlation with the viral titer (Figure 3B). Interestingly, when we studied the correlation between svRNA 1 and 2 levels and SERINC5 mRNA levels, we found an inverse correlation that was significant for svRNA 1 and svRNA 2 in both sample types (Figure 3C).

Altogether, these data indicated that in SARS-CoV-2-infected patients, the level of SERINC5 mRNA was reduced and this reduction was inversely proportional to the viral titer and the level of svRNA 1 and svRNA 2.

### 3.2. Levels of SARS-CoV-2 svRNAs 1 and 2 are inversely correlated with the levels of SERINC5 in Vero E6 and HEK293T-hACE2 infected cells

**Figure 4:**
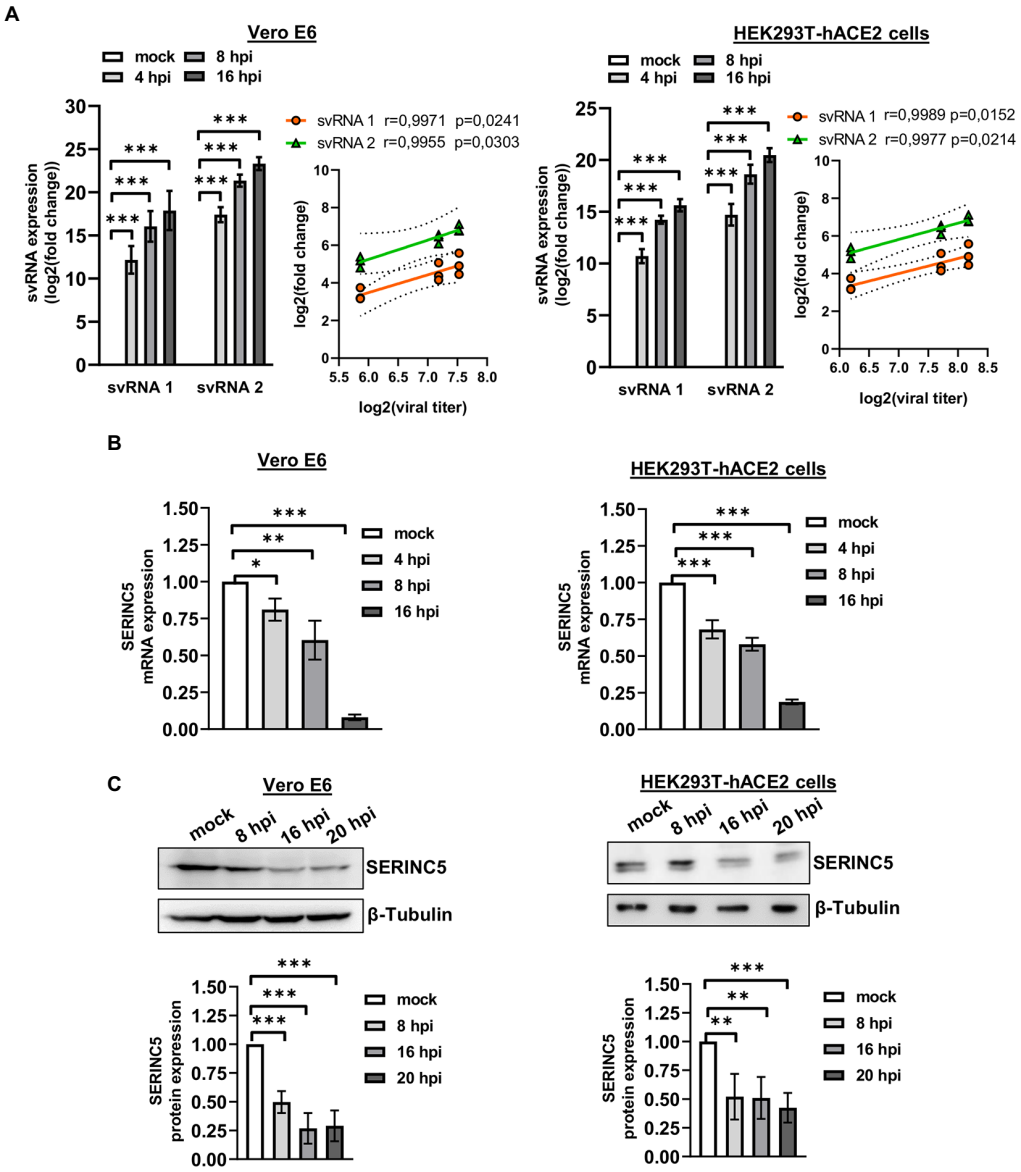
Analysis of the expression of svRNAs 1 and 2 and SERINC5 in Vero E6 and HEK293T-hACE2 infected cells. **(A)** RT-qPCR analysis of svRNAs 1 and 2 expression and correlation analysis between log2 fold changes obtained for svRNA 1 and svRNA 2 and log 2 viral titers in SARS-CoV-2-infected (MOI, 1 PFU/cell) Vero E6 (left panels) and HEK293T-hACE2 (right panels) cells at 4, 8, and 16 hpi. The ΔΔCt method was used for relative quantification, with U6 snRNA as an endogenous control. Data are represented as log2 fold change with respect to mock values and are the mean ± SD of at least three independent experiments. Viral titer was expressed as E mRNA copies/mL sample. **(B)** RT-qPCR analysis of SERINC5 mRNA expression in SARS-CoV-2-infected (MOI, 1 PFU/cell) Vero E6 (left panel) and HEK293T-hACE2 (right panel) cells at 4, 8, and 16 hpi. The ΔΔCt method was used for relative quantification with RPP30 mRNA as an endogenous control. Data are represented in logarithmic scale as fold change with respect to mock values and are the mean ± SD of at least three independent experiments. **(C)** Western blot analysis of SERINC5 protein in SARS-CoV-2-infected (MOI, 1 PFU/cell) Vero E6 (left panels) and HEK293T-hACE2 (right panels) cells at 8, 16, and 20 hpi. Blots are representative of at least three independent experiments. The scatter plot shows the densitometric analysis of the protein normalized to β-tubulin and represented as fold change relative to mock. Differences from mock values were found to be statistically significant at *p < 0.05, **p < 0.01, and ***p < 0.001.

After the identification of SARS-CoV-2 svRNAs in patient samples and demonstrating an inverse correlation with SERINC5 expression, we performed similar studies *in vitro* using the cell lines Vero E6 and HEK293T-hACE2 (Figure 4). To that end, Vero E6 and HEK293T-hACE2 cells were infected with SARS-CoV-2 with a MOI of 1 PFU/cell and at 4, 8, and 16 h post-infection (hpi) the expression of svRNAs 1 y 2 and SERINC5 mRNA was analyzed by RT-qPCR. Based on the RT-qPCR data, we found that both svRNAs progressively accumulated in both cell lines and this accumulation directly correlated with the viral titer (Figure 4A). Interestingly, when the levels of SERINC5 mRNA were analyzed, we detected a progressive reduction throughout the infection until extremely low values at 16 hpi in both cell lines (Figure 4B), which was inversely correlated with the virus titer. To confirm this reduction, the expression of SERINC5 at the protein level was also analyzed by western blot. A clear reduction in the levels of SERINC5 protein throughout the infection was detected, with their lowest values at 16 and 20 hpi in both cell lines (Figure 4C). These results indicated that the infection of SARS-CoV-2 reduces the level of SERINC5, that this reduction is inversely proportional to the levels of SARS-CoV-2 svRNAs 1 and 2, and that this effect is independent of the cell type and species.

### 3.3. svRNAs 1 and 2 can bind the 3’UTR of SERINC5 mRNA and reduce SERINC5 expression *in vitro*

**Figure 5:**
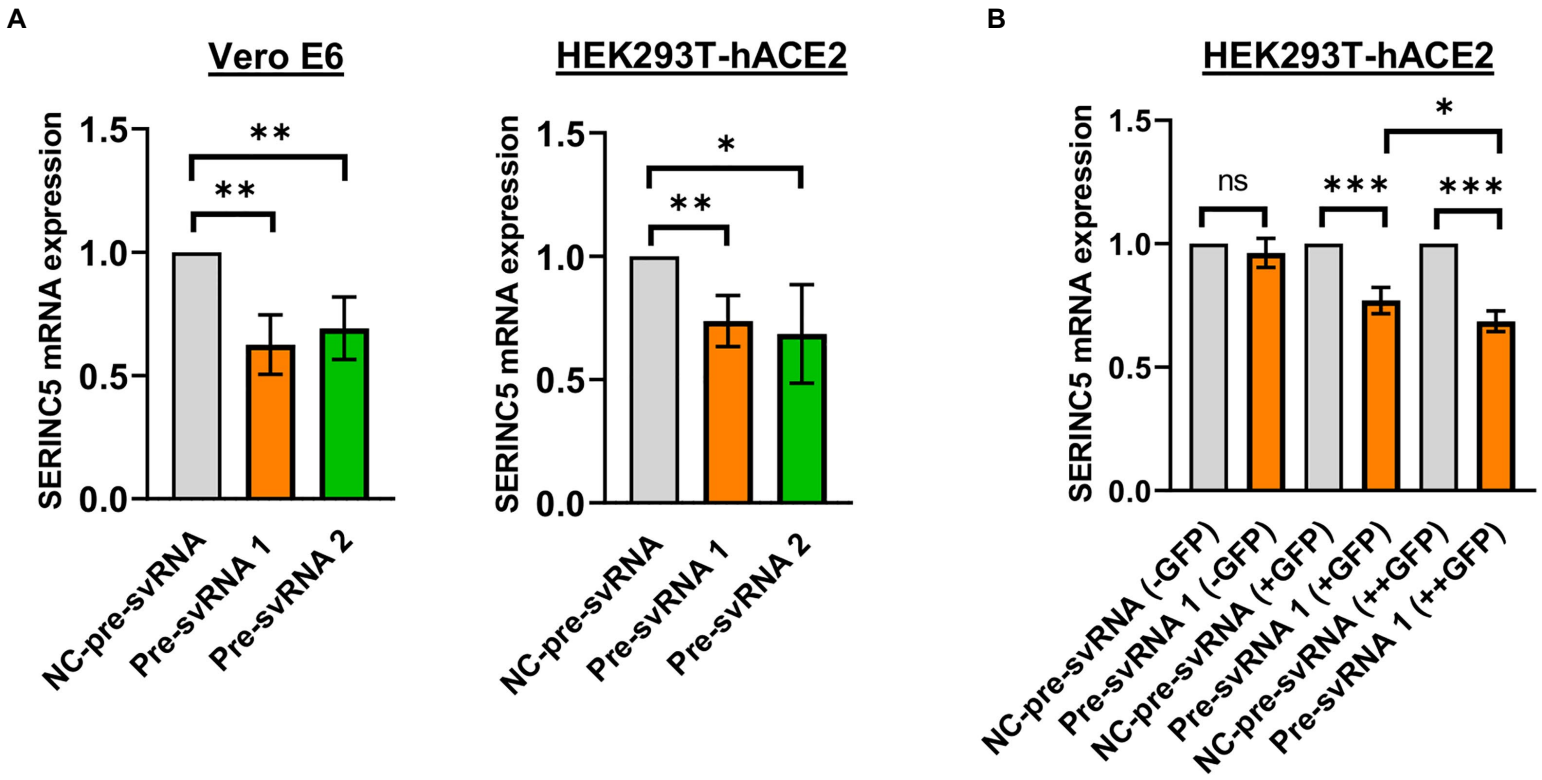
Analysis of the levels of SERINC5 mRNA in Vero E6 and HEK293T-hACE2 cells transfected with svRNAs 1 and 2 mimics. **(A)** RT-qPCR analysis of SERINC5 mRNA level in Vero E6 (left panel) and HEK293T-hACE2 (right panel) cells transfected with an oligonucleotide that mimics the precursor of svRNA 1 or 2 (Pre-svRNA 1 and 2, respectively) or an irrelevant precursor svRNA (NC-pre-svRNA) as a negative control. Data are represented as fold change with respect to values from control samples. **(B)** RT-qPCR analysis of SERINC5 mRNA level in HEK293T-hACE2 cells co-transfected with Pre-svRNA 1 and a GFP-expressing plasmid. The study was performed on these cells after sorting them into three populations, non-expressing (−GFP), intermediate expressing (+GFP), and highly expressing (++GFP) GFP cells. Data are represented as fold change with respect to values from negative control-transfected cells.

Once the existence of svRNA 1 and 2 during SARS-CoV-2 infection was demonstrated and the levels of these svRNAs inversely correlated with the level of SERINC5, we explored the ability of these svRNAs to post-transcriptionally regulate endogenous SERINC5 expression. To that end, Vero E6 and HEK293T-hACE2 cells were transfected with svRNA 1 or svRNA 2 mimic molecules (Pre-svRNA 1 and 2) and the levels of SERINC5 mRNA were determined by RT-qPCR. As shown in Figure 5A, a clear reduction in SERINC5 mRNA levels was observed in both Vero E6 and HEK293T-hACE2 cells transfected with either of the two Pre-svRNAs, confirming that both svRNAs are responsible for the downregulation of SERINC5 observed during SARS-CoV-2 infection. Then, we analyzed whether the svRNAs effect was dose-dependent. To this end, we co-transfected HEK293T-hACE2 cells with a GFP-expressing plasmid and the svRNA 1 mimic, and then sorted the cells into three populations: non-expressing, moderately expressing, and highly expressing GFP. The analysis of these three populations showed that the expression of SERINC5 was inversely proportional to the expression of GFP, confirming that, at least for svRNA 1, the level of expression of this svRNA mimic within the cell influences the expression of SERINC5 in a dose-dependent way (Figure 5B).

**Figure 6:**
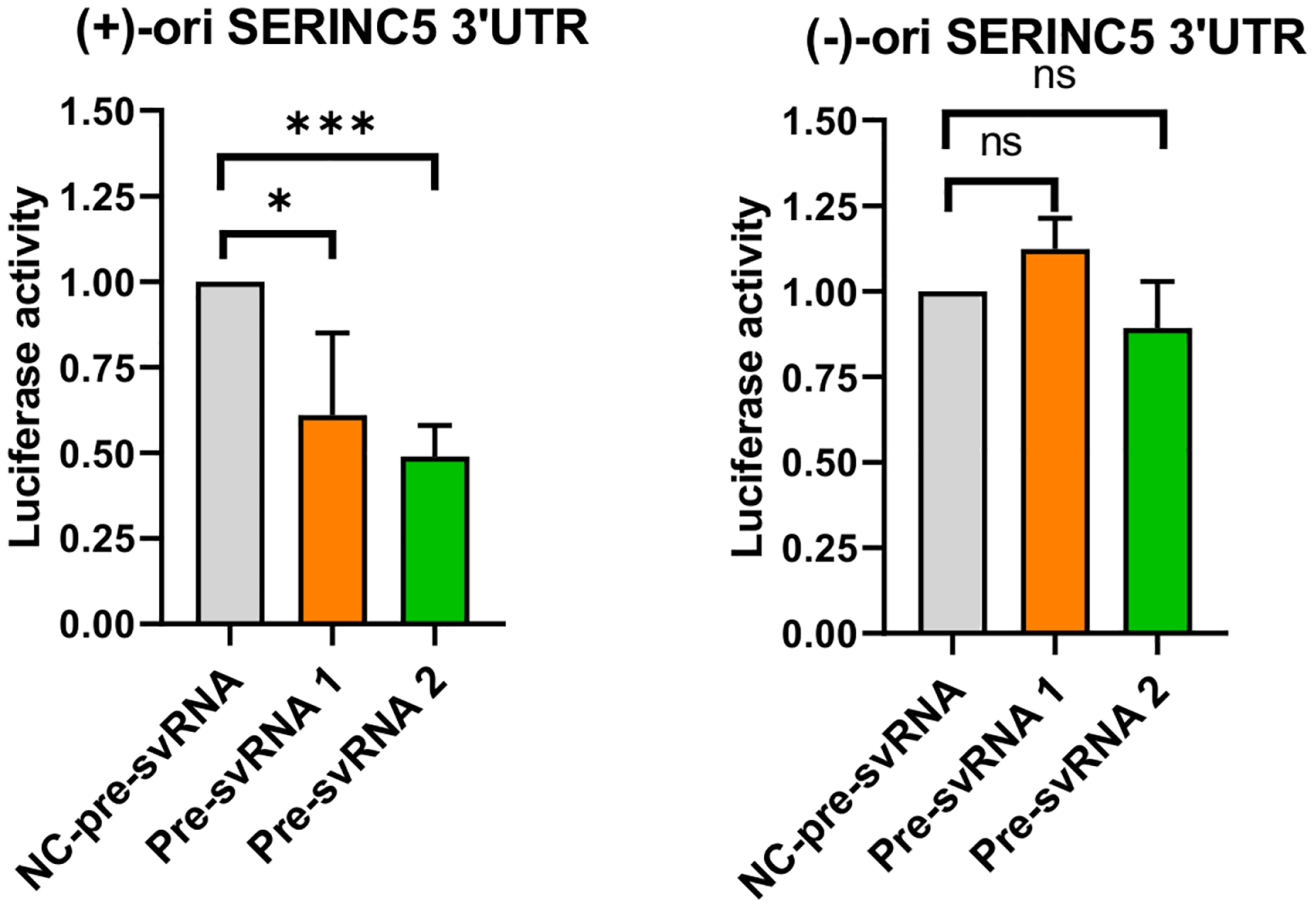
Study of the binding capacity of svRNA 1 and 2 to the 3’UTR of SERINC5 mRNA. Vero E6 cells were co-transfected with a negative control oligonucleotide (NC-pre-svRNA), the oligonucleotide mimic of svRNA 1 or 2 (pre-svRNA 1 and 2) together with the reporter constructs containing the Firefly Luciferase gene fused with the SERINC5-3′UTR in the direct (+) (left panel) or reverse (−) direction (right panel), and the Renilla Luciferase control vector. At 48 h post-transfection the luciferase activity was analyzed and the data obtained were normalized to the levels of Renilla Luciferase (Control of transfection). Differences from control values were found to be statistically significant at *p < 0.05, **p < 0.01, and ***p < 0.001.

Finally, we evaluated the ability of svRNAs 1 and 2 to bind to the 3′UTR of SERINC5 mRNA target using a luciferase reporter assay. To this end, we cloned a portion of the 3′UTR of SERINC5 mRNA downstream of the Firefly Luciferase reporter gene in the pMIR plasmid in direct (+) or reverse (−) direction [pMIR-Luc-SERINC5-3’UTR(+) and pMIR-Luc-SERINC5-3’UTR(−), respectively]. Then, we co-transfected each plasmid into Vero E6 cells together with the control plasmid expressing Renila Luciferase and the respective svRNA mimic or its negative control. As shown in Figure 6, co-transfection of the wild-type SERINC5 3′UTR reporter [pMIR-Luc-SERINC5-3’UTR(+)] with svRNAs 1 or 2 mimics reduced significantly the luciferase activity when compared with the mimic control transfected cells (Figure 6, left panel), whereas no effect was observed when the reporter carried the 3′UTR cloned in the reverse direction [pMIR-Luc-SERINC5-3’UTR(−)] (Figure 6, right panel). Altogether, these data indicate that svRNAs 1 and 2 function as a miRNA-like regulators of SERINC5, directly targeting the 3′UTR of the SERINC5 mRNA.

### 3.4. Treatment with antisense oligonucleotides against svRNA 1 and 2 recovers SERINC5 expression and reduces the levels of SARS-CoV-2  N and S viral proteins

**Figure 7:**
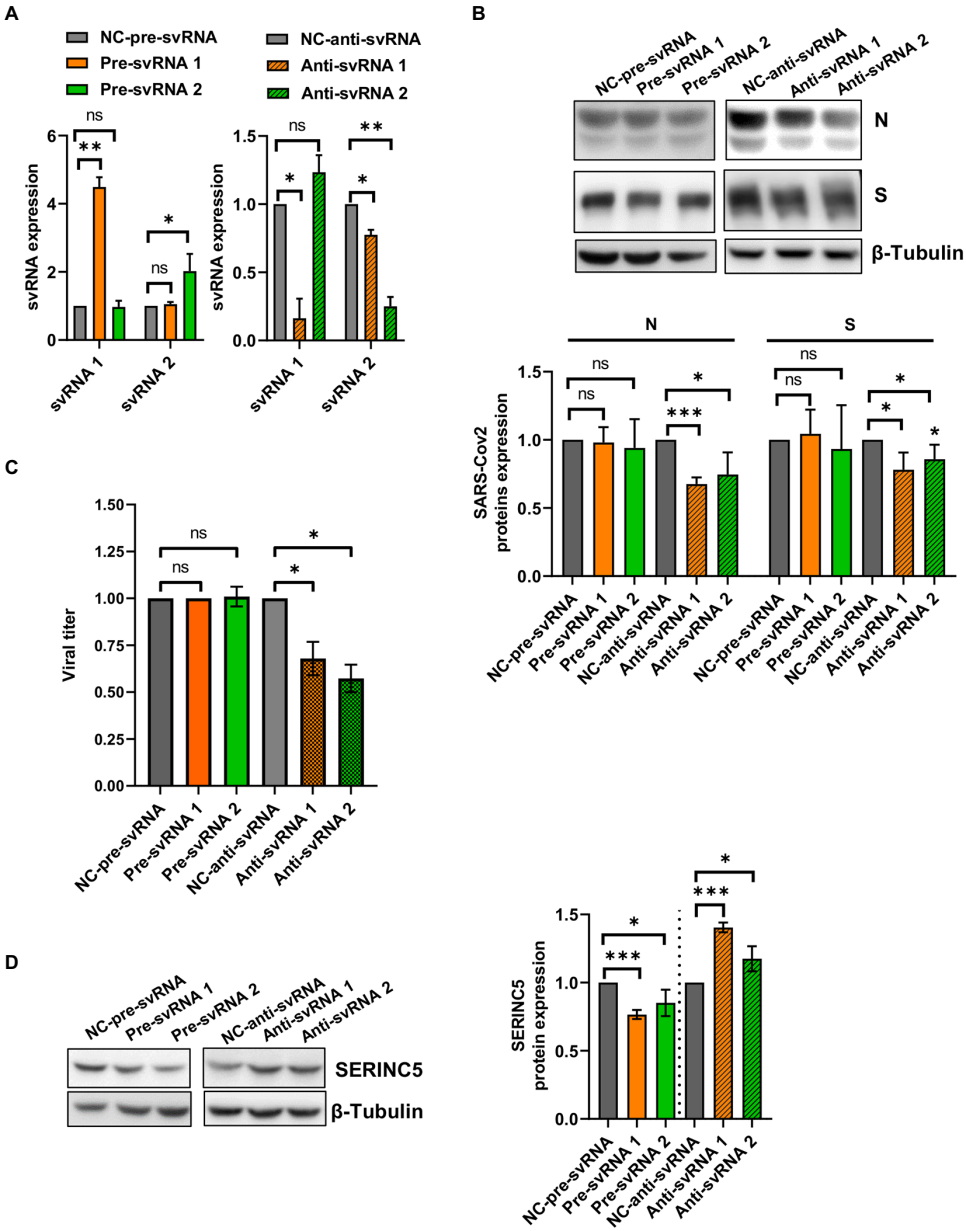
Expression levels of svRNAs 1 and 2, viral proteins and cellular SERINC5 in Vero E6 cells treated with mimic or anti-sense molecules for svRNA 1 and 2 and infected with SARS-CoV-2. Vero E6 cells were transfected with specific oligonucleotides mimic for svRNA 1 or svRNA 2 (Pre-svRNA 1 and 2), or specific antisense oligonucleotides for svRNA 1 or svRNA 2 (Anti-svRNA 1 and 2), and at 36 h post-transfection the cells were infected with SARS-CoV-2 (1 PFU/cell). The levels of svRNAs, N, S and SERINC5 proteins were analyzed at 20 hpi and the virus production in the cell supernatants at 24 hpi. **(A)** RT-qPCR analysis of svRNAs 1 and 2 levels in cells transfected with Pre-svRNA 1 and 2 (left panel), or Anti-svRNA 1 and 2 (right panel). Data are represented as fold change respect to the negative control (NC-pre-svRNA or NC-anti-svRNA)-transfected and SARS-CoV-2-infected cells (control samples). The control value represents the mean of all control samples. **(B)** Western blot analysis of viral N and S proteins. The scatter plot shows the densitometric analysis of N and S proteins normalized to β-tubulin and represented as fold change relative to negative control-transfected cells. The control value represents the mean of all control samples. **(C)** Virus production. The viral titers in the cell supernatants were determined at 24 hpi by plaque assay. Data are represented as fold change respect to the negative control (NC-pre-svRNA or NC-anti-svRNA)-transfected and SARS-CoV-2-infected cells (control samples). **(D)** Western blot analysis of SERINC5. The scatter plot shows the densitometric analysis of SERINC5 protein normalized to β-tubulin and represented as fold change relative to SARS-CoV-2-infected, negative control-transfected cells. The control value represents the mean of all control samples. Differences from negative control values were found to be statistically significant at *p < 0.05, **p < 0.01 and ***p < 0.001.

To explore the biological function of svRNAs 1 and 2, we performed overexpression or silencing experiments. To that end, Vero E6 cells were transfected with specific mimic (overexpression) or anti-svRNA (silencing) oligonucleotides (Table 1). At 36 h post-transfection, the cells were infected with SARS-CoV-2 at a MOI of 1 PFU/cell and at 20 hpi the levels of svRNAs, viral N and S proteins, and SERINC5 were analyzed. Previously, we confirmed the overexpression and silencing of svRNAs 1 and 2 by RT-qPCR. Levels of svRNA 1 and 2 were increased 4- and 2-fold, respectively, in pre-svRNA 1 or 2-transfected cells compared to cells transfected with a non-related mimic sequence [negative control (NC-Pre-svRNA)] (Figure 7A, left panel). Conversely, they were reduced by almost 75% in anti-svRNA 1 and 2-transfected cells compared to cells transfected with a non-related sequence inhibitor [negative control (NC) anti-miR] (Figure 7A, right panel). Interestingly, in cells where svRNA 1 had been silenced we also noted a moderate reduction of svRNA 2 level, suggesting that svRNA 1 biogenesis positively regulates the generation of svRNA 2. After that, we analyzed the effect of the mimic svRNAs and anti-svRNAs on virus replication by analyzing the expression of N and S proteins by western blot (Figure 7B). No significant differences were found in the levels of S and N proteins when cells were treated with mimic molecules. In contrast, when cells were treated with the anti-svRNAs a significant reduction in the levels of S and N proteins was detected, being more evident in the case of N protein (40% reduction; Figure 7B). In addition, we also analyzed the effect of the mimic svRNA and anti-svRNA molecules on virus production (24 hpi). No significant differences were detected between cells treated or untreated with the mimic molecules. In contrast, a very moderate reduction was observed in cells treated with anti-svRNA 1 (1.50 fold decrease) or 2 (1.75 fold decrease), which could be in concordance with the reduction observed in the levels of N and S proteins (Figure 7C). Although these differences are very low, they are statistically significant.

Finally, we explored by Western-blot whether the overexpression or silencing of SARS-CoV-2 svRNAs affected the levels of SERINC5 protein (Figure 7D). As expected, we observed that the treatment with either svRNA 1 or svRNA 2 mimics reduced SERINC5 expression while anti-svRNA 1 and 2 treatments achieved a moderate increase in SERINC5 levels compared to NC-transfected cells.

Altogether, these data confirm the regulation of SERINC5 by the SARS-CoV-2 svRNAs and indicate that anti-svRNA 1 and 2 have only a moderate effect on virus production, possibly by increasing the levels of SERINC5, the target of the viral svRNAs.

### 3.5. Anti-svRNAs treatment modifies the levels of MAVS in SARS-CoV-2 infected cells

A novel antiviral activity of SERINC5 has been recently described (Zeng et al., 2021). SERINC5 has been shown to translocate to the mitochondrial membrane after viral infection, where it associates with MAVS and promotes its oligomerization. Aggregated MAVS acts as a central hub for signal transduction, leading to changes in the expression of several genes involved in inflammation, apoptosis and cell cycle as part of the cellular response against viral infection (Zhang et al., 2020; Zeng et al., 2021). However, there are several viruses, including SARS-CoV-2, that are capable of controlling the MAVS cascade by establishing interactions between the elements of this cascade and several viral proteins. This strategy allows them to escape and over-activate the innate immune response during the course of infection (Fu et al., 2021; Fung et al., 2021; Han et al., 2021; Liu et al., 2021; Zotta et al., 2021; Li et al., 2022; Thorne et al., 2022; Zheng et al., 2022).

In a first approach, we evaluated the control of SERINC5 on the levels of endogenous MAVS protein in uninfected Vero E6 cells, by examining its levels in cells that overexpress SERINC5. We found that these cells exhibited a 5-fold increase of endogenous MAVS protein (Supplementary Figure S2). Furthermore, we observed, as described before (Zeng et al., 2021), that SERINC5 partially co-localized with mitochondria (Supplementary Figure S3) and with MAVS (Supplementary Figure S4) in Vero E6 cells.

**Figure 8:**
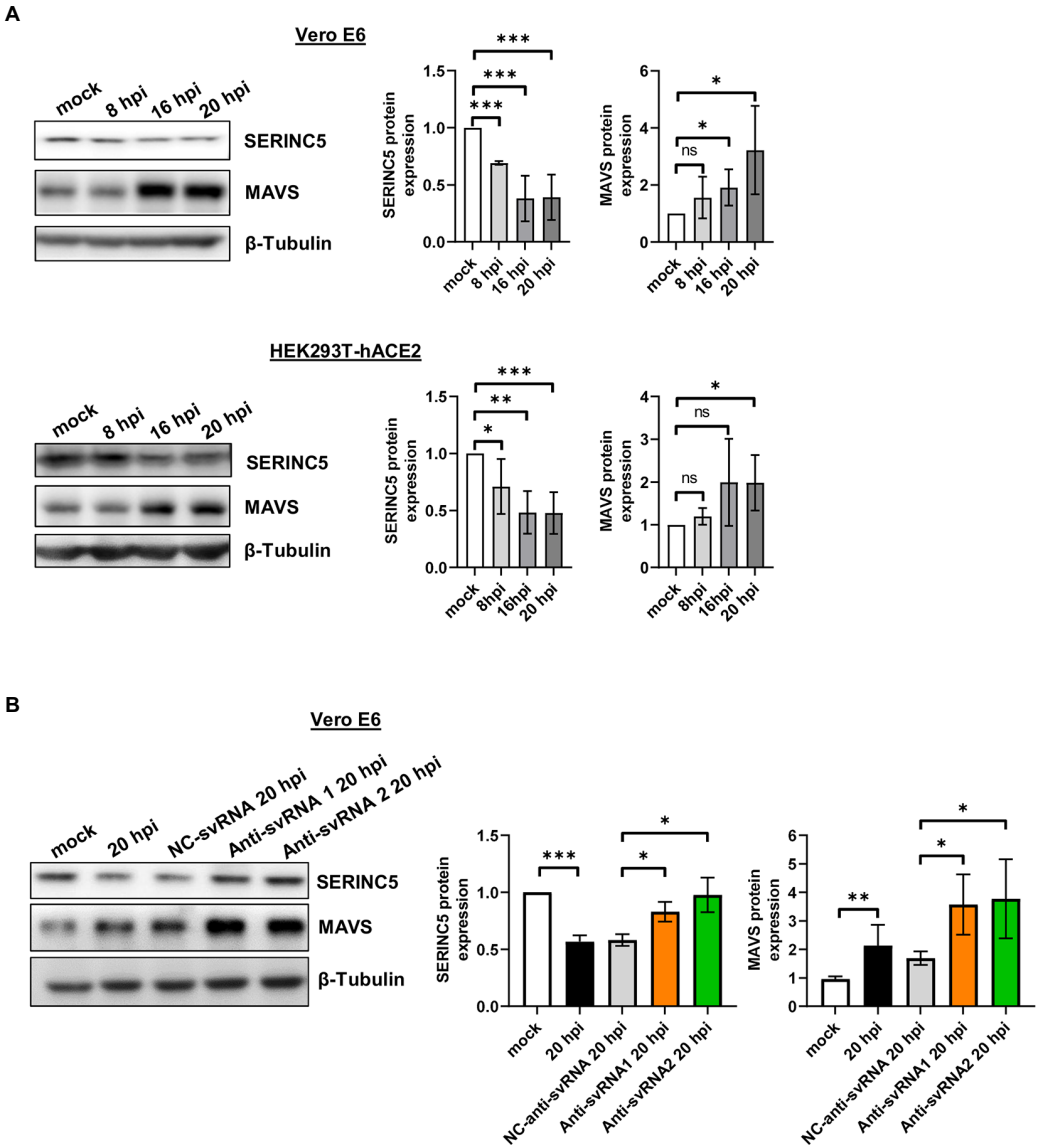
Analysis of MAVS expression during SARS-CoV-2 infection of Vero E6 and HEK293T-hACE2 cells treated or not with anti-sense molecules for svRNA 1 and 2. **(A)** Western blot analysis of SERINC5 and MAVS in Vero E6 (left) or HEK293T-hACE2 cells (right) mock-infected or infected with SARS-CoV-2 (MOI of 1 PFU/cell) at 8, 16, and 20 hpi. The scatter plot shows the densitometric analysis of MAVS protein normalized to β-tubulin and represented as fold change relative to non-infected (mock) cells. **(B)** Western blot analysis of MAVS in Vero E6 cells mock-transfected or transfected with anti-svRNA 1, anti-svRNA 2, or NC-anti-svRNA for 36 h and then infected with SARS-CoV-2 with a MOI of 1PFU/cell for 20 h. The scatter plot shows the densitometric analysis of MAVS and SERINC5 proteins normalized to β-tubulin, represented as fold change relative to non-transfected mock-infected (mock) cells. Differences from control values were found to be statistically significant at *p < 0.05, **p < 0.01, and ***p < 0.001.

Based on this regulation and considering that SARS-CoV-2 infection of Vero E6 and HEK293T-hACE2 cells triggers a decrease in SERINC5 protein levels, a reduction in MAVS protein levels would be expected under these conditions. To verify this, Vero E6 and HEK293T-hACE2 cells were infected with SARS-CoV-2 (MOI of 1 PFU/cell) and the expression of SERINC5 and MAVS was analyzed at 8, 16, and 20 hpi by Western blot. Surprisingly, in both cell types the levels of MAVS protein increased when those of SERINC5 decreased during viral infection (Figure 8A), suggesting the involvement of a SERINC5-independent and positive regulatory mechanism of MAVS. In this line, it has been reported that the viral protein nsp5 increases the stability of MAVS by promoting its SUMOylation and, consequently, increasing its levels (Li et al., 2021). Based on our findings, although both SERINC5-dependent and -independent mechanisms coexist during the infection, positive regulation of MAVS by the SERINC5-independent mechanism apparently has a greater effect on MAVS expression than the negative effect of reducing SERINC5 (SERINC5-dependent mechanism).

Then we explored whether anti-svRNA 1 and 2 treatments had any effect on MAVS expression. In particular, we examined the levels of MAVS protein after SARS-CoV-2 infection of Vero E6 cells transfected with the anti-svRNA 1 or 2 and we compared them with those of NC-anti-svRNA-transfected cells. As shown in Figure 8B, the increase in MAVS levels after SARS-CoV-2 infection was significantly reinforced by the anti-svRNA treatments. This reflects an accumulative effect on MAVS by the SERINC5-dependent pathway when SERINC5 is recovered.

### 3.6. Anti-svRNA 1 treatment lowers the induction of innate immune-related genes

**Figure 9:**
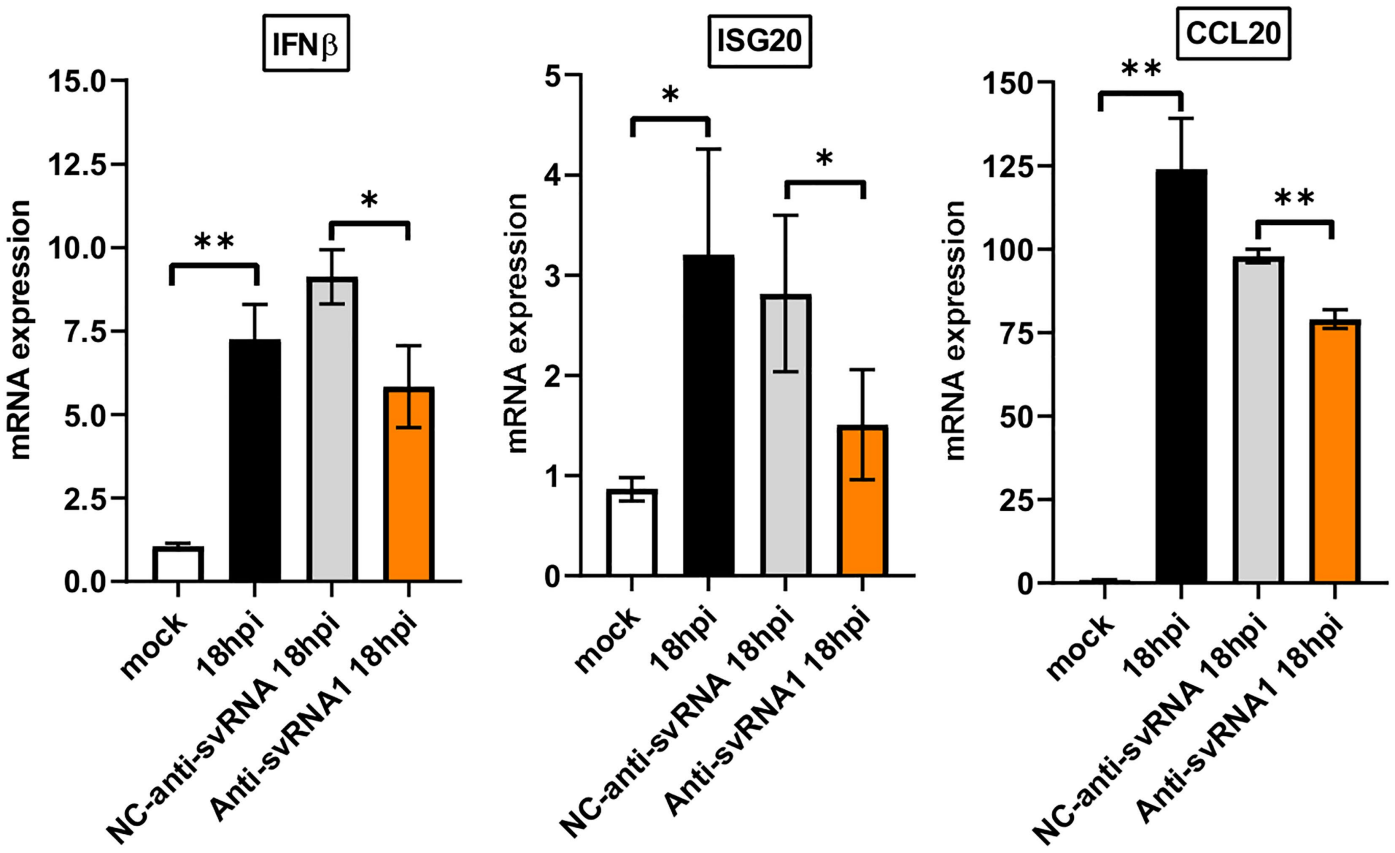
Expression levels of IFNβ, ISG20, and CCL20 mRNAs in Vero E6 cells treated or not with anti-sense molecules for svRNA 1. Vero E6 cells were mock transfected or transfected with either a specific antisense oligonucleotide for svRNA 1 (Anti-svRNA1) or an irrelevant oligonucleotide (NC-anti-svRNA) as a negative control. At 36 h post-transfection the cells were infected with SARS-CoV-2 (1 PFU/cell) and the levels of IFNβ, ISG20 and CCL20 mRNAs were analyzed at 18 hpi by RT-qPCR. The ΔΔCt method was used for relative quantification with RPP30 mRNA as an endogenous control. Data are represented as fold change relative to non-transfected mock-infected (mock) cells and are the mean ± SD of at least three independent experiments. Differences from control values were found to be statistically significant at *p < 0.05 and **p < 0.01.

SERINC5 has recently been considered as a key factor in the innate immune response (Firrito et al., 2018; Li et al., 2020; Zeng et al., 2021). Since we observed that the anti-svRNA treatment recovers the expression of SERINC5 in Vero E6 cells infected with SARS-CoV-2 (Figure 7D), we evaluate the expression of certain innate immune related genes under these conditions. In particular, we analyzed by RT-qPCR the mRNA levels of interferon β (IFNβ), an interferon-stimulated gene [interferon stimulated exonuclease gene 20 (ISG20)] and a chemokine [chemokine (C-C motif) ligand 20 (CCL20)] after the SARS-CoV-2 infection of VeroE6 cells previously transfected with anti-svRNA 1, the most efficient svRNA inhibitor recovering SERINC5 (Figure 7D), or with the negative control (NC) anti-svRNA The oligonucleotides used are described in Table 1. We found that SARS-CoV-2 infection induced the mRNA expression of IFNβ, ISG20, and CCL20 and that this induction was partially counteracted when the cells recover SERINC5 expression by treatment with anti-svRNA 1 (Figure 9). These data suggests that SERINC5 is a negative regulator of the innate immune response. To confirm this negative role of SERINC5, the expression of IFNβ, ISG20, and CCL20 was evaluate in VeroE6 cells over-expressing SERINC5 in comparison to control cells. We found that VeroE6 cells over-expressing SERINC5 showed reduced levels of IFNβ, ISG20, and CCL20 mRNAs when compared to control cells (Supplementary Figure S5). Overall, our results suggest that recovery of SERINC5 by anti-svRNA treatment lowers the host innate response triggered by the virus.

## 4. Discussion

SERINC5 is an unconventional restriction and cell-associated innate immunity factor required to restrict the infectivity of certain viruses such as HIV-1, MLV, simian immunodeficiency virus (SIV), EIAV, vesicular stomatitis virus (VSV), and Zika virus (ZIKV) (Rosa et al., 2015; Chande et al., 2016; Heigele et al., 2016; Timilsina et al., 2020; Zeng et al., 2021). These studies have shown that when SERINC5 is over-expressed within the cell, it effectively suppresses the viral particle infectivity. It incorporates into nascent viral particles and compromises the formation of the virus-cell fusion pore for viral entry into new target cells. Recent work has demonstrated that SERINC5 operates in the same way during SARS-CoV-2 infection of Calu3 cells (Timilsina et al., 2022). SERINC5 inhibits SARS-CoV-2 infection by binding to SARS-CoV-2 S protein, thus blocking the fusion step during virus entry. Furthermore, two additional roles have recently been attributed to this protein. In the case of the Hepatitis B virus (HBV), SERINC5 inhibits virion secretion by interfering with the glycosylation of HBV envelope proteins (10). In contrast, in HIV-1, VSV, and ZIKV, SERINC5 inhibits virus infection by interacting with the outer mitochondrial membrane protein MAVS facilitating its aggregation and, consequently, triggering the activation of downstream signaling pathways (Zeng et al., 2021).

In many of the viruses described above, different mechanisms to counteract the antiviral effect of SERINC5 have been described. HIV-1, MLV, and EIA encode viral proteins to counteract SERINC5 activities (Usami et al., 2015; Ahi et al., 2016; Chande et al., 2016). In SARS-CoV-2 (Timilsina et al., 2022), it has been demonstrated that protein 7a counteracts SERINC5 by blocking SERINC5 incorporation in budding virions and by forming a complex with SERINC5 and SARS-CoV-2 S protein, hindering the activity of the SERINC5 molecules incorporated in budding virions. Interestingly, these studies with SARS-CoV-2 were performed in Calu3 cells at 2 and 6 hpi, a period in which authors did not observed changes in SERINC5 mRNA levels. A similar result was also obtained here at 4 hpi in the *in silico* study using GSE148729 data for that cell line, where no major changes in the mRNA level were detected (Supplementary Figure S1). However, we revealed in the *in silico* study that levels of SERINC5 mRNA started to decline from 12hpi. Moreover, we have confirmed that reduction of SERINC5 mRNA levels during SARS-Cov2 infection also occurred in VeroE6 and HEK293T-hACE2 cells (Figure 4), mainly at late stage, and in COVID-19 patient samples (Figure 1).Therefore, these data suggest that SARS-CoV-2 infection counteracts SERINC5 protein activity by protein 7a in the first stage and SERINC5 mRNA/protein levels at late stage by repression in the expression of this gene, highlighting the importance of the host factor in virus restriction.

Here, we demonstrated that levels of SERINC5 were reduced during the infection by SARS-CoV-2 both in cell cultures and in COVID-19 patients. Since no viral protein capable of repressing the expression of SERINC5 in host cells was identified, we hypothesized that svRNAs encoded in the SARS-CoV-2 genome could be responsible for this repression during infection. Through an *in silico* study, we identified two viral small RNAs (svRNAs 1 and 2) with predicted binding sites in the 3’UTR region of the SERINC5 gene. svRNA 1 is a 24 nt-long RNA, located in the intergenic sequence between N and ORF10 genes at the 3’end of the SARS-CoV-2 genome, that showed a sequence similar to a region of the cellular microRNA precursor pre-miRNA-431. Interestingly, this conservation is maintained among several mammalian species, including humans. As described for other viral miRNAs with high homologies to host miRNAs in seed sequence, this strategy could allow the virus to mislead host cells or take control of pre-existing regulatory pathways of host miRNAs (Gottwein et al., 2007; Skalsky et al., 2007; Kincaid et al., 2012). On the other hand, svRNA 2 is a 24 nt-long RNA located in the N gene. Both svRNAs exhibited high conservation grades among different coronaviruses and showed *in silico-*binding capacity to the 3’UTR of SERINC5 mRNA.

First, we proved the existence of both svRNAs in nasopharyngeal and saliva samples from COVID-19 patients and SARS-CoV-2-infected cell lines. In both cases, the level of SERINC5 mRNA was reduced and this reduction was inversely proportional to the viral titer and to the level of svRNA 1 and svRNA 2. This effect was independent of the cell type and species according to the experiments with Vero E6 and HEK293T-hACE2 cells. Moreover, we explored whether SARS-CoV-2 svRNA production was dependent on the cellular miRNA pathway by the analysis of svRNAs expression in HEK293T-hACE2 cells, in which Dicer and Argonaute 2 (Ago2) proteins were silenced (Supplementary Figure S6). We found that knocking down the expression of Dicer and Ago2 did not significantly affect the expression of either svRNA in infected cells (Supplementary Figure S6). These data are consistent with the previous results reported in SARS-CoV (Morales et al., 2017) and suggest that svRNAs are generated by alternative pathway/s.

Then we demonstrated that both svRNAs down-regulate the levels of endogenous SERINC5 mRNA and that this regulation occurs directly through the binding of the svRNAs to the 3’UTR. This regulation was demonstrated by overexpression and silencing experiments. Overexpression of svRNA 1 or svRNA 2 in cells infected with SARS-CoV-2, promoted the reduction of SERINC5 protein levels, and their partial silencing with antisense oligonucleotide against these svRNAs, partially recovers SERINC5 expression. In these conditions, SERINC5 recovery was accompanied by a reduction in the levels of SARS-CoV-2 N and S proteins and by a very moderate decrease in virus production. This slight impact on viral production is possible because the anti-svRNAs effect on viral proteins did not reach compromised levels in these cell lines in which virus infection and replication are very favorable.

Then we showed that SERINC5 controlled the levels of MAVS in uninfected Vero E6 cells, as SERINC5 overexpression (~2-fold) triggered a marked increase in MAVS protein (~5-fold). This phenomenon was previously observed in the HEK293T cells (Zeng et al., 2021). However, during infection in Vero E6 and HEK293T-hACE2 cells, even though SERINC5 levels were reduced by the action of svRNAs, the levels of MAVS did not decrease in parallel. On the contrary, they progressively increased, suggesting that a SERINC5-independent mechanism would be responsible for the augmentation in MAVS during infection. In this line, recent work has shown that the SARS-CoV-2 nsp5 protein increases the stability of MAVS by promoting its SUMOylation (Li et al., 2021). As MAVS increases during infection, the positive regulation by this SERINC5-independent mechanism apparently prevails over the negative effect of reducing SERINC5 (SERINC5-dependent mechanism). On the other hand, we have observed that the recovery of SERINC5 by anti-svRNA treatment during infection was accompanied by a greater increase in MAVS than that normally induced by SARS-CoV-2 infection alone, thus showing an accumulative effect. Altogether, these data suggest that SARS-CoV-2 expresses svRNAs to block host control over MAVS by reducing SERINC5 expression and favoring control of MAVS by viral proteins such as nsp5. Through treatment with anti-svRNAs, we were able to restore the SERINC5-dependent regulation of MAVS and reduce the levels of SARS-CoV-2 viral proteins N and S.

Different studies have described how SARS-CoV-2 proteins nsp5, N, M, ORF6, ORF9b, and ORF10 interfere with IFN production by targeting components of RIG1/MDA5-MAVS-IFN signaling pathways (Fu et al., 2021; Fung et al., 2021; Han et al., 2021; Liu et al., 2021; Zotta et al., 2021; Li et al., 2022; Thorne et al., 2022; Zheng et al., 2022). These findings suggest that SARS-CoV-2 proteins exert important control over the MAVS cascade. The interactions between those viral proteins and components of the MAVS cascade could condition the activity of MAVS in different pathways during the course of the infection, facilitating the evasion of the virus from the innate immune response and favoring its pathogenicity (Mattoo et al., 2022).

The innate immune system functions as the first line of defense against SARS-CoV-2; however, dysregulated innate immune responses can induce aberrant inflammation, cytokine storm, tissue damage, and acute respiratory distress syndrome in the host (Karki and Kanneganti, 2022). We found that anti-svRNA 1 treatment, which recovers SERINC5, lowers the induction of innate immune-related genes (IFNβ, ISG20 and CCL20; Figure 9; Supplementary Figure S5), indicating that SERINC5 acts as a negative regulator of these genes during SARS-CoV-2 infection. Overall, our data suggest that anti-svRNA treatment partially mitigates the innate immune response and promotes the reestablishment of basal levels of these immune signals. Although we still need further experiments to assess the therapeutic role of the use of antisense oligonucleotides against these svRNAs, our findings highlight potential therapeutic targets based on their action on key genes of the innate immune response.

Finally, considering that svRNAs 1 and 2 were easily detected in COVID-19 patient samples and that their levels correlated well with SARS-CoV-2 viral titer, svRNAs could be good candidate biomarkers in the diagnosis of the disease. A higher cohort of COVID-19 patient samples will allow testing of this possibility.

## Data availability statement

The datasets presented in this study can be found in online repositories. The names of the repository/repositories and accession number(s) can be found in the article/Supplementary material.

## Ethics statement

The studies involving human participants were reviewed and approved by Ethics Committee of Hospital Universitario de la Ribera (Valencia, Spain) and performed under the guidelines set forth by the Declaration of Helsinki. The patients/participants provided their written informed consent to participate in this study.

## Author contributions

SM, EE, FA, and FI designed the study. SM, FA, M-PR, OC-R, and BP-B performed the experiments. BL constructed the Luciferase reporter plasmids. RP-M, SR-G, and FG-G performed the in silico reanalysis of deposited sequencing data. OM-M and AC provided the samples of COVID-19 patients. SM, FA, and EE wrote the paper. All authors reviewed the manuscript.

## Funding

This work has been supported by grant CSIC-COV19-106 (202020 E164) from the Spanish National Research Council (CSIC) to FA and FI, and grant from RTI2018-101291-B-I00 to EE from the Spanish Ministry of Science and Innovation.

## Conflict of interest

The authors declare that the research was conducted in the absence of any commercial or financial relationships that could be construed as a potential conflict of interest.

## Publisher’s note

All claims expressed in this article are solely those of the authors and do not necessarily represent those of their affiliated organizations, or those of the publisher, the editors and the reviewers. Any product that may be evaluated in this article, or claim that may be made by its manufacturer, is not guaranteed or endorsed by the publisher.
