# Supplementary material for: SARS-CoV-2-encoded small RNAs are able to repress the host expression of SERINC5 to facilitate viral replication
Source: Front Microbiol. 2023 Feb 16;14:1066493. doi: 10.3389/fmicb.2023.1066493 (PMC9978209; doi:10.3389/fmicb.2023.1066493)
Supplement: Supplementary file 3 [file Data_Sheet_1.PDF]

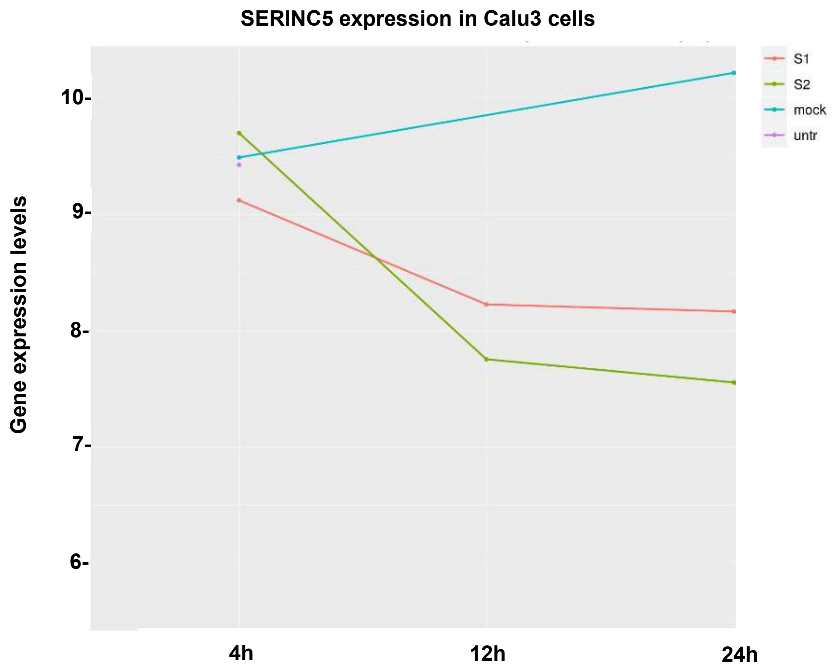

Figure S1. Reanalysis of the GSE148729 dataset to determine the SERINC5 mRNA expression during the infection of Calu3 cells with SARS-CoV and SARS-CoV-2. Expression levels of SERINC5 mRNA after reanalysis of poly A-RNA-sequencing data from Calu3 cells infected with SARS-CoV (S1), SARS-CoV-2 (S2), mock-infected or untreated (untr) for 4, 12 and 24 h (GSE148729 dataset).

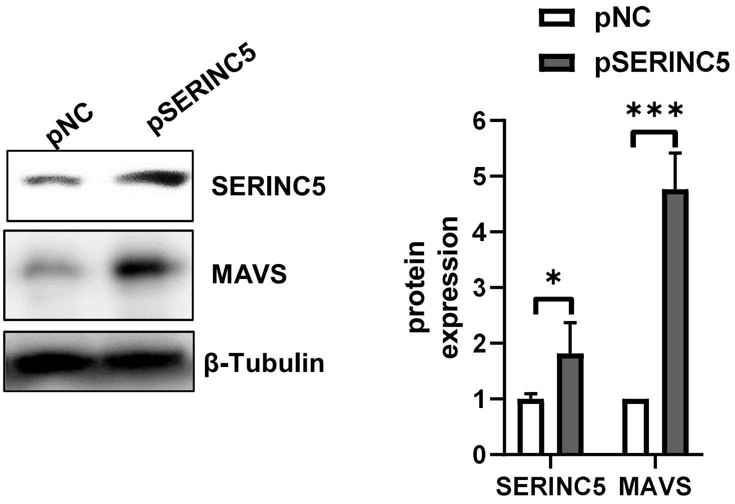

Figure S2. Levels of MAVS in Vero E6 cells over-expressing SERINC5. Western blot analysis of SERINC5 and MAVS in Vero E6 cells overexpressing SERINC5 in parallel with a GFP marker (pSERINC5) with respect to control cells expressing only the GFP marker (pNC). Scatter plot shows the densitometric analysis of SERINC5 and MAVS normalized to β-tubulin and represented as fold change relative to control cells. Differences from control values were found to be statistically significant at $*p < 0.05$ and $***p < 0.001$.

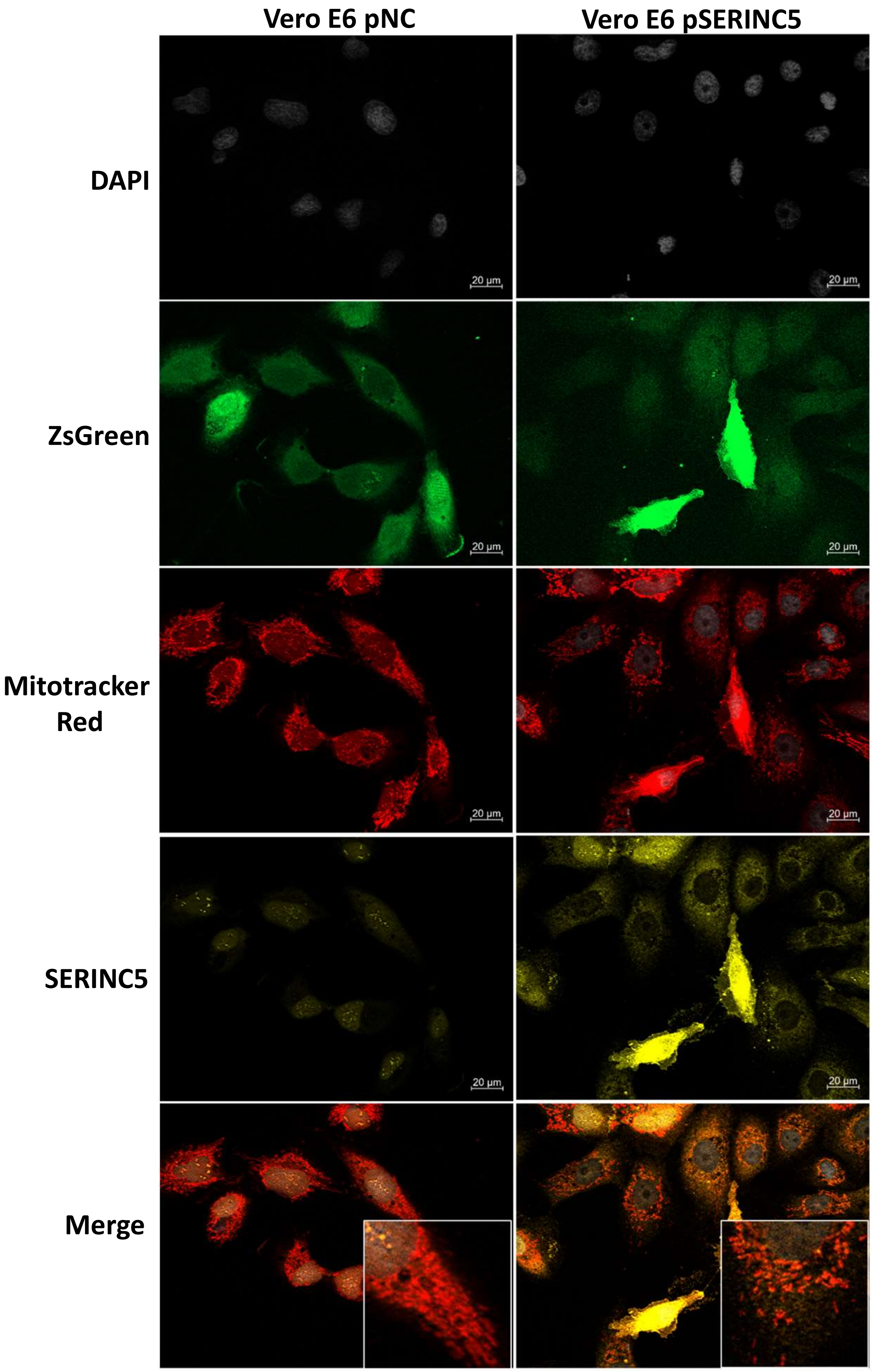

Figure S3. Analysis of the subcellular localization of SERINC5 in Vero E6 cells overexpressing SERINC5 by Immunofluorescence. Mitochondria from Vero E6 cells transfected with the empty plasmid pIRES2 ZsGreen1 (Vero pNC) or the plasmid pIRES2 ZsGreen1 containing the *Chlorocebus* SERINC5 cDNA (Vero pSERINC5) were stained with Mitotracker Red (red) for 30min. Cells were then fixed and incubated with Anti-SERINC5 (yellow) to detect SERINC5, mounted with Prolong Gold antifade reagent with DAPI to stain nuclei (gray), and images were taken with Apotome-equipped Axio Observer Z1 microscope (Carl Zeiss AG). ZsGreen signal indicated cells that incorporated the constructs. Merge images were obtained from the respective Mitotracker Red and Anti-SERINC5 images. Scale bars, 20 µm.

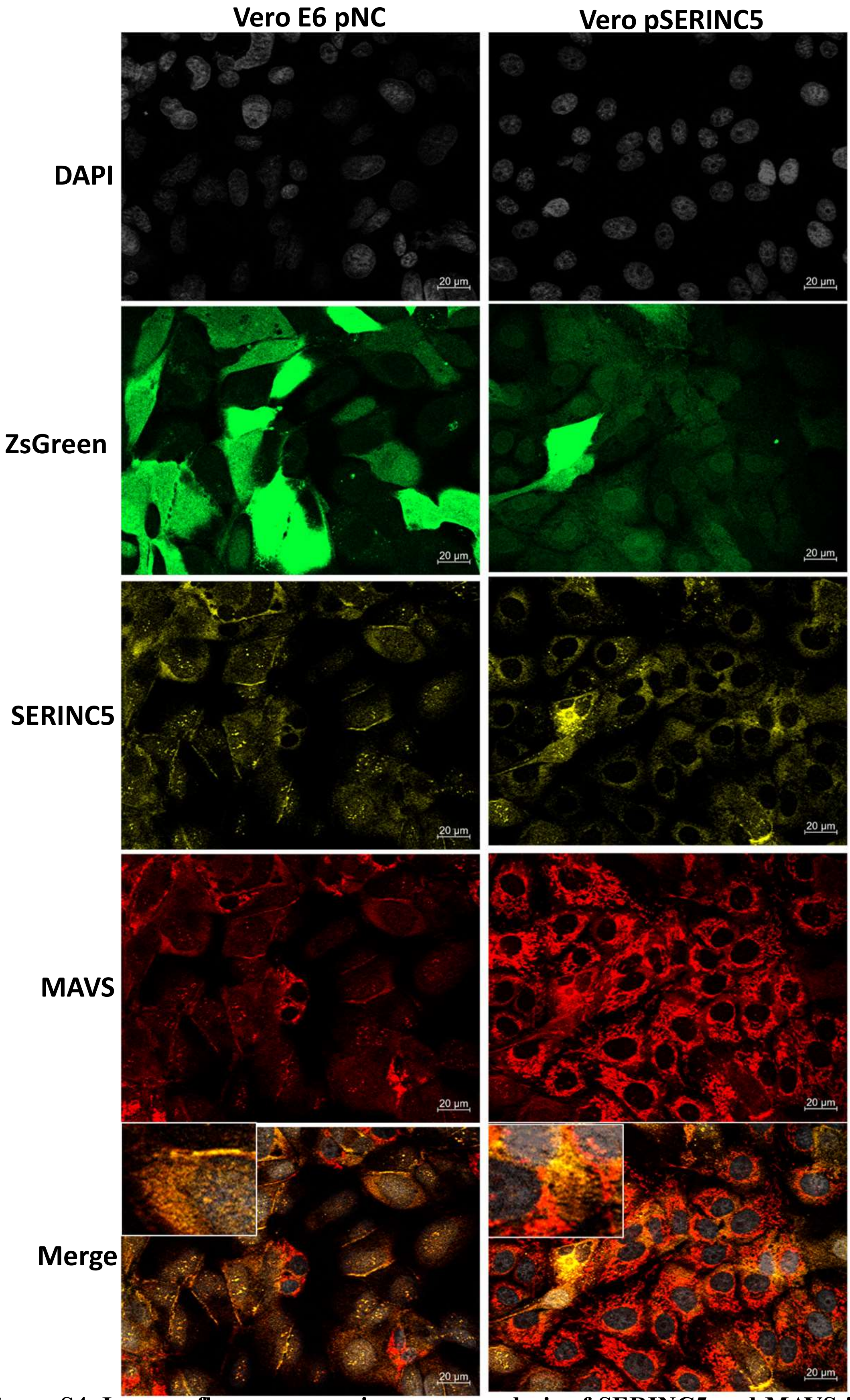

Figure S4. Immunofluorescence microscopy analysis of SERINC5 and MAVS in Vero E6 cells overexpressing SERINC5. Vero E6 cells transfected with the empty plasmid pIRES2 ZsGreen1 (Vero pNC) or the plasmid pIRES2 ZsGreen1 containing the *Chlorocebus* SERINC5 cDNA (Vero pSERINC5) were fixed and incubated with Anti-SERINC5 (yellow) to detect SERINC5 and with Anti-MAVS (red) to detect MAVS. Then, they were mounted with Prolong Gold antifade reagent with DAPI to stain nuclei (gray), and images were taken with Apotome-equipped Axio Observer Z1 microscope (Carl Zeiss AG). ZsGreen signal indicated cells that incorporated the constructs. Merge images were obtained from the respective Anti-SERINC5 and Anti-MAVS images. Scale bars, 20 µm.

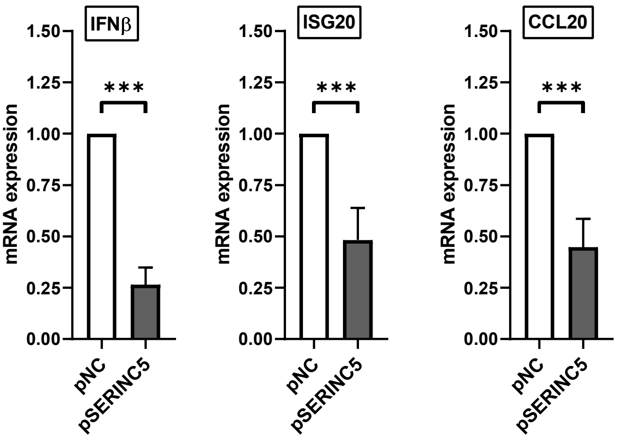

Figure S5. mRNA levels of IFNβ, ISG20 and CCL20 in Vero E6 cells over-expressing SERINC5. RT-qPCR analysis of IFNβ, ISG20 and CCL20 mRNA levels in Vero E6 cells over-expressing SERINC5 (pSERINC5). The ΔΔCt method was used for relative quantification with RPP30 mRNA as an endogenous control. Data are represented as fold change with respect to values from Vero E6 cells containing the empty plasmid (pNC) and are the mean ±SD of at least three independent experiments. Differences from control values were found to be statistically significant at ***$p < 0.001$.

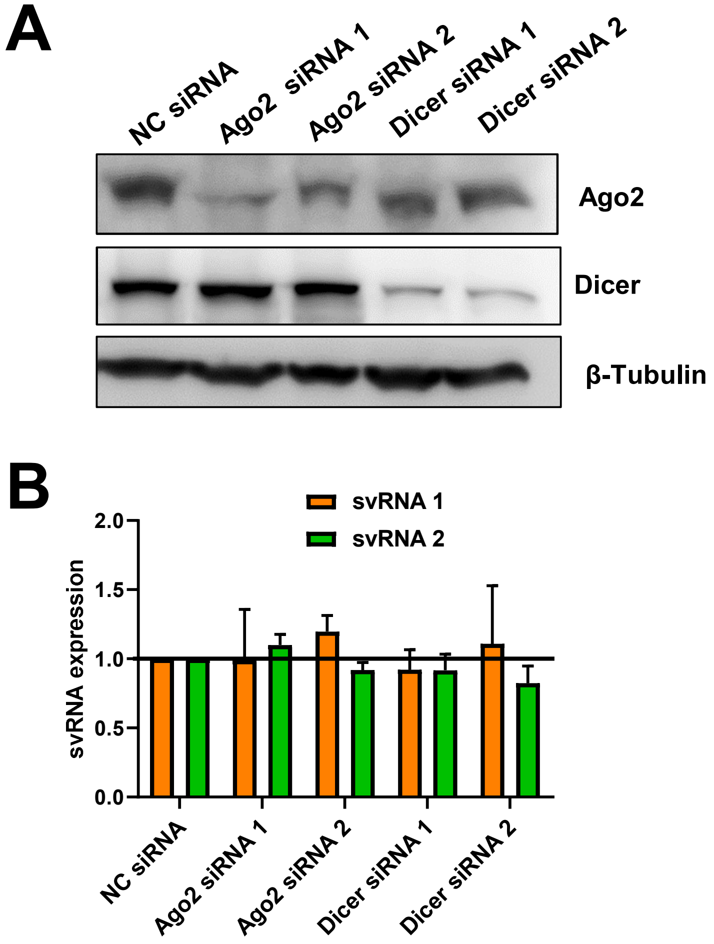

Figure S6. Levels of svRNAs in Dicer- and Ago2- silenced HEK293T-hACE2 cells. (A) Western-blot analysis of Ago2 and Dicer expression in HEK293T-hACE2 cells transfected for 36 h with Ago2 siRNA 1, Ago2 siRNA 2, Dicer siRNA 1, Dicer siRNA 2, or the negative control (NC-siRNA). **(B)** RT-qPCR analysis of the expression of svRNA 1 and 2 in HEK293T-hACE2 cells transfected for 36 h with Ago2 siRNA 1, Ago2 siRNA 2, Dicer siRNA 1, Dicer siRNA 2, or the negative control (NC-siRNA), and infected with SARS-CoV-2 for 18 h (MOI 1 PFU/cell). Data are represented as fold change with respect to the NC-siRNA transfected and SARS-CoV-2-infected cells. Differences from control values were found to be statistically non-significant.
